# Supplementary material for: Cardiovascular–kidney–metabolic syndrome and all-cause and cardiovascular mortality: A retrospective cohort study
Source: PLoS Med. 2025 Jun 26;22(6):e1004629. doi: 10.1371/journal.pmed.1004629 (PMC12200875; doi:10.1371/journal.pmed.1004629)
Supplement: S1 Table — (DOCX) [file pmed.1004629.s001.docx]

# Table S1. Restricted mean survival time regression model for all-cause and CVD mortality by cardiovascular–kidney–metabolic syndrome stage

|  | All-cause mortality | | | | |  |  |  |  |
| --- | --- | --- | --- | --- | --- | --- | --- | --- | --- |
| CKM | N | n of deaths | HR* | (95% CI) | | RMST* | RMST difference | (95% CI) | |
| Stage 0 | 147,024 | 3,002 | Ref. |  |  | 23.395 |  |  |  |
| Stage 1 | 100,585 | 3,280 | 0.96 | (0.91 | ,1.02) | 23.248 | -0.147 | (-0.19 | ,-0.11) |
| Stage 2 | 238,647 | 22,469 | 1.36 | (1.30 | ,1.42) | 23.140 | -0.255 | (-0.29 | ,-0.22) |
| Stage 3 | 9,925 | 6,415 | 2.13 | (2.02 | ,2.25) | 21.161 | -2.234 | (-2.44 | ,-2.03) |
| Stage 4 | 19,421 | 6,423 | 2.37 | (2.25 | ,2.49) | 19.043 | -4.352 | (-4.72 | ,-3.98) |
| All CKM† | 368,578 | 38,587 | 1.33 | (1.28 | ,1.39) |  |  |  |  |
|  |  |  |  |  |  |  |  |  |  |
| Zero components | 251,564 | 6,750 |  |  |  | 23.159 |  |  |  |
| One component | 146,826 | 11,065 | 1.21 | (1.17 | ,1.26) | 23.291 | 0.132 | 0.09 | 0.17 |
| Two components | 56,647 | 8,772 | 1.49 | (1.43 | ,1.54) | 22.841 | -0.318 | (-0.40 | ,-0.24) |
| Three components | 40,047 | 7,279 | 1.57 | (1.51 | ,1.63) | 22.719 | -0.440 | (-0.54 | ,-0.34) |
| Four components | 16,001 | 5,406 | 2.12 | (2.03 | ,2.21) | 21.484 | -1.675 | (-1.90 | ,-1.45) |
| Five components | 4,517 | 2,317 | 3.53 | (3.34 | ,3.72) | 18.764 | -4.394 | (-4.91 | ,-3.88) |
| Increase by one component |  |  | 1.22 | (1.21 | ,1.23) |  |  |  |  |
|  | CVD mortality | | | | |  |  |  |  |
| CKM | N | n of deaths | HR* | (95% CI) | | RMST* | RMST difference | (95% CI) | |
| Stage 0 | 147,024 | 282 | Ref. |  |  | 23.914 |  |  |  |
| Stage 1 | 100,585 | 368 | 1.13 | (0.95 | ,1.35) | 23.847 | -0.068 | (-0.08 | ,-0.05) |
| Stage 2 | 238,647 | 4,631 | 2.89 | (2.51 | ,3.32) | 23.811 | -0.104 | (-0.12 | ,-0.09) |
| Stage 3 | 9,925 | 1,594 | 5.27 | (4.51 | ,6.16) | 22.549 | -1.365 | (-1.48 | ,-1.25) |
| Stage 4 | 19,421 | 1,950 | 7.42 | (6.40 | ,8.60) | 20.449 | -3.465 | (-3.69 | ,-3.24) |
| All CKM† | 368,578 | 8,543 | 2.81 | (2.45 | ,3.22) |  |  |  |  |
|  |  |  |  |  |  |  |  |  |  |
| Zero components | 251,564 | 761 |  |  |  | 23.808 |  |  |  |
| One component | 146,826 | 2,190 | 2.02 | (1.84 | ,2.22) | 23.841 | 0.033 | 0.02 | 0.05 |
| Two components | 56,647 | 2,084 | 2.84 | (2.58 | ,3.13) | 23.656 | -0.152 | (-0.18 | ,-0.12) |
| Three components | 40,047 | 1,816 | 3.17 | (2.88 | ,3.50) | 23.553 | -0.255 | (-0.30 | ,-0.21) |
| Four components | 16,001 | 1,429 | 4.54 | (4.10 | ,5.02) | 22.810 | -0.998 | (-1.11 | ,-0.89) |
| Five components | 4,517 | 545 | 6.68 | (5.88 | ,7.58) | 21.045 | -2.763 | (-3.06 | ,-2.47) |
| Increase by one component | |  | 1.37 | (1.35 | ,1.40) |  |  |  |  |

*The HR and RMST were adjusted for age, sex, educational levels, smoking status, drinking status, and physical activity groups.

†All CKM does not include stage 0.

Abbreviations: CKM: cardiovascular–kidney–metabolic syndrome; CVD: cardiovascular disease; RMST: restricted mean survival time; HR: hazard ratio; CI: confidence interval; Ref: reference group.
