# Supplementary material for: Cardiovascular–kidney–metabolic syndrome and all-cause and cardiovascular mortality: A retrospective cohort study
Source: PLoS Med. 2025 Jun 26;22(6):e1004629. doi: 10.1371/journal.pmed.1004629 (PMC12200875; doi:10.1371/journal.pmed.1004629)
Supplement: S2 Table — (DOCX) [file pmed.1004629.s002.docx]

# Table S2. Hazard ratios for all-cause and CVD mortality stratified by cardiovascular–kidney–metabolic syndrome stage (Multiple imputation imputed for missing information)

|  | All-cause mortality | | | | |
| --- | --- | --- | --- | --- | --- |
| CKM | N | n of deaths | HR* | (95% CI) | |
| Stage 0 | 162,297 | 3,188 | Ref. |  |  |
| Stage 1 | 106,247 | 3,394 | 0.97 | (0.92 | ,1.02) |
| Stage 2 | 245,547 | 22,817 | 1.36 | (1.31 | ,1.42) |
| Stage 3 | 10,010 | 6,469 | 2.16 | (2.05 | ,2.28) |
| Stage 4 | 19,960 | 6,569 | 2.39 | (2.28 | ,2.52) |
| All CKM† | 381,764 | 39,249 | 1.33 | (1.28 | ,1.39) |
|  |  |  |  |  |  |
| Zero components | 273,120 | 7,076 |  |  |  |
| One component | 151,899 | 11,294 | 1.22 | (1.18 | ,1.26) |
| Two components | 57,619 | 8,881 | 1.49 | (1.44 | ,1.55) |
| Three components | 40,714 | 7,384 | 1.57 | (1.52 | ,1.63) |
| Four components | 16,172 | 5,470 | 2.13 | (2.04 | ,2.22) |
| Five components | 4,537 | 2,332 | 3.54 | (3.36 | ,3.74) |
| Increase by one component |  |  | 1.22 | (1.21 | ,1.23) |
|  | CVD mortality | | | | |
| CKM | N | n of deaths | HR* | (95% CI) | |
| Stage 0 | 162,297 | 296 | Ref. |  |  |
| Stage 1 | 106,247 | 378 | 1.14 | (0.95 | ,1.35) |
| Stage 2 | 245,547 | 4,691 | 2.90 | (2.53 | ,3.33) |
| Stage 3 | 10,010 | 1,613 | 5.38 | (4.62 | ,6.27) |
| Stage 4 | 19,960 | 1,990 | 7.55 | (6.53 | ,8.73) |
| All CKM† | 381,764 | 8,672 | 2.81 | (2.46 | ,3.21) |
|  |  |  |  |  |  |
| Zero components | 273,120 | 790 | Ref. |  |  |
| One component | 151,899 | 2,232 | 2.04 | (1.86 | ,2.24) |
| Two components | 57,619 | 2,107 | 2.87 | (2.61 | ,3.16) |
| Three components | 40,714 | 1,848 | 3.23 | (2.93 | ,3.56) |
| Four components | 16,172 | 1,442 | 4.56 | (4.12 | ,5.04) |
| Five components | 4,537 | 549 | 6.76 | (5.96 | ,7.67) |
| Increase by one component |  |  | 1.37 | (1.35 | ,1.40) |

*The HR were adjusted for age, sex, educational levels, smoking status, drinking status, and physical activity groups.

†All CKM does not include stage 0.

Abbreviations: CKM: cardiovascular–kidney–metabolic syndrome; CVD: cardiovascular disease; HR: hazard ratio; CI: confidence interval; Ref: reference group
