# Supplementary material for: Cardiovascular–kidney–metabolic syndrome and all-cause and cardiovascular mortality: A retrospective cohort study
Source: PLoS Med. 2025 Jun 26;22(6):e1004629. doi: 10.1371/journal.pmed.1004629 (PMC12200875; doi:10.1371/journal.pmed.1004629)
Supplement: S3 Table — (DOCX) [file pmed.1004629.s003.docx]

# Table S3. Hazard ratios for all-cause and CVD mortality stratified by cardiovascular–kidney–metabolic syndrome stage (additionally considering the use of analgesics and cancer history)

|  | All-cause mortality | | | | |  | HR2 additionally considering the use of  analgesics and cancer history | | |
| --- | --- | --- | --- | --- | --- | --- | --- | --- | --- |
| CKM | N | n of deaths | HR1* | (95% CI) | |  | HR2* | (95% CI) | |
| Stage 0 | 147,024 | 3,002 | Ref. |  |  |  | Ref. |  |  |
| Stage 1 | 100,585 | 3,280 | 0.96 | (0.91 | ,1.02) |  | 0.96 | (0.91 | ,1.02) |
| Stage 2 | 238,647 | 22,469 | 1.36 | (1.30 | ,1.42) |  | 1.36 | (1.30 | ,1.42) |
| Stage 3 | 9,925 | 6,415 | 2.13 | (2.02 | ,2.25) |  | 2.14 | (2.02 | ,2.26) |
| Stage 4 | 19,421 | 6,423 | 2.37 | (2.25 | ,2.49) |  | 2.36 | (2.24 | ,2.48) |
| All CKM† | 368,578 | 38,587 | 1.33 | (1.28 | ,1.39) |  | 1.33 | (1.28 | ,1.39) |
|  |  |  |  |  |  |  |  |  |  |
| Zero components | 251,564 | 6,750 |  |  |  |  | Ref. |  |  |
| One component | 146,826 | 11,065 | 1.21 | (1.17 | ,1.26) |  | 1.22 | (1.17 | ,1.26) |
| Two components | 56,647 | 8,772 | 1.49 | (1.43 | ,1.54) |  | 1.49 | (1.43 | ,1.54) |
| Three components | 40,047 | 7,279 | 1.57 | (1.51 | ,1.63) |  | 1.57 | (1.51 | ,1.63) |
| Four components | 16,001 | 5,406 | 2.12 | (2.03 | ,2.21) |  | 2.11 | (2.03 | ,2.20) |
| Five components | 4,517 | 2,317 | 3.53 | (3.34 | ,3.72) |  | 3.53 | (3.34 | ,3.72) |
| Increase by one component |  |  | 1.22 | (1.21 | ,1.23) |  | 1.22 | (1.21 | ,1.23) |
|  | CVD mortality | | | | |  |  | | |
| CKM | N | n of deaths | HR1* | (95% CI) | |  | HR2* | (95% CI) | |
| Stage 0 | 147,024 | 282 | Ref. |  |  |  | Ref. |  |  |
| Stage 1 | 100,585 | 368 | 1.13 | (0.95 | ,1.35) |  | 1.14 | (0.96 | ,1.36) |
| Stage 2 | 238,647 | 4,631 | 2.89 | (2.51 | ,3.32) |  | 2.90 | (2.52 | ,3.33) |
| Stage 3 | 9,925 | 1,594 | 5.27 | (4.51 | ,6.16) |  | 5.30 | (4.53 | ,6.19) |
| Stage 4 | 19,421 | 1,950 | 7.42 | (6.40 | ,8.60) |  | 7.44 | (6.42 | ,8.63) |
| All CKM† | 368,578 | 8,543 | 2.81 | (2.45 | ,3.22) |  | 2.82 | (2.46 | ,3.24) |
|  |  |  |  |  |  |  |  |  |  |
| Zero components | 251,564 | 761 |  |  |  |  | Ref. |  |  |
| One component | 146,826 | 2,190 | 2.02 | (1.84 | ,2.22) |  | 2.02 | (1.84 | ,2.22) |
| Two components | 56,647 | 2,084 | 2.84 | (2.58 | ,3.13) |  | 2.84 | (2.58 | ,3.12) |
| Three components | 40,047 | 1,816 | 3.17 | (2.88 | ,3.50) |  | 3.18 | (2.89 | ,3.51) |
| Four components | 16,001 | 1,429 | 4.54 | (4.10 | ,5.02) |  | 4.53 | (4.09 | ,5.01) |
| Five components | 4,517 | 545 | 6.68 | (5.88 | ,7.58) |  | 6.69 | (5.90 | ,7.60) |
| Increase by one component |  |  | 1.37 | (1.35 | ,1.40) |  | 1.37 | (1.35 | ,1.40) |

*The HR were adjusted for age, sex, educational levels, smoking status, drinking status, and physical activity groups.

†All CKM does not include stage 0.

Abbreviations: CKM: cardiovascular–kidney–metabolic syndrome; CVD: cardiovascular disease; HR: hazard ratio; CI: confidence interval; Ref: reference group
