# Supplementary material for: Cardiovascular–kidney–metabolic syndrome and all-cause and cardiovascular mortality: A retrospective cohort study
Source: PLoS Med. 2025 Jun 26;22(6):e1004629. doi: 10.1371/journal.pmed.1004629 (PMC12200875; doi:10.1371/journal.pmed.1004629)
Supplement: S4 Table — (1) Hazard ratios for risks of cause-specific mortality and end-stage kidney disease stratified by number and type of cardiovascular–kidney–metabolic syndrome component. (2) Hazard ratios for risks of cause-specific mortality and end-stage kidney disease stratified by number and type of cardiovascular–kidney–metabolic syndrome component. (DOCX) [file pmed.1004629.s004.docx]

# Table S4-1. Hazard ratios for risks of cause-specific mortality and end-stage kidney disease stratified by number and type of cardiovascular–kidney–metabolic syndrome component

|  |  |  |  | All-cause mortality | | | | CVD mortality | | | | HD mortality | | | | Stroke mortality | | | |
| --- | --- | --- | --- | --- | --- | --- | --- | --- | --- | --- | --- | --- | --- | --- | --- | --- | --- | --- | --- |
|  |  | N | (%) | Deaths | HR | (95% CI) | | Deaths | HR | (95% CI) | | Deaths | HR | (95% CI) | | Deaths | HR | (95% CI) | |
| Zero components |  | 251,564 | (48.8) | 6,750 | Ref. |  |  | 761 | Ref. |  |  | 174 | Ref. |  |  | 242 | Ref. |  |  |
| One component | Hypertension only | 82,929 | (16.1) | 7,240 | 1.28 | (1.23 | ,1.33) | 1,618 | 2.40 | (2.18 | ,2.65) | 387 | 2.49 | (2.04 | ,3.06) | 591 | 2.80 | (2.36 | ,3.31) |
|  | CKD only | 13,921 | (2.7) | 1,394 | 1.46 | (1.37 | ,1.56) | 196 | 1.63 | (1.36 | ,1.95) | 48 | 1.86 | (1.30 | ,2.65) | 57 | 1.38 | (0.98 | ,1.95) |
|  | Diabetes only | 2,915 | (0.6) | 441 | 1.86 | (1.67 | ,2.06) | 72 | 2.42 | (1.84 | ,3.18) | 24 | 3.10 | (1.87 | ,5.14) | 24 | 2.41 | (1.48 | ,3.91) |
|  | Metabolic syndrome only | 609 | (0.1) | 53 | 1.19 | (0.89 | ,1.58) | 10 | 1.88 | (0.97 | ,3.63) | 3 | 2.84 | (0.90 | ,8.92) | 2 | 0.66 | (0.09 | ,4.68) |
|  | Hyperlipidemia only | 46,452 | (9.0) | 1,937 | 0.97 | (0.92 | ,1.02) | 294 | 1.34 | (1.16 | ,1.56) | 75 | 1.42 | (1.05 | ,1.92) | 72 | 1.01 | (0.75 | ,1.35) |
| Two components | Hypertension+CKD | 10,427 | (2.0) | 3,424 | 1.93 | (1.84 | ,2.03) | 916 | 3.99 | (3.56 | ,4.47) | 205 | 3.92 | (3.10 | ,4.97) | 336 | 4.49 | (3.68 | ,5.47) |
|  | Hypertension+Diabetes | 2,054 | (0.4) | 489 | 1.99 | (1.80 | ,2.21) | 100 | 3.24 | (2.55 | ,4.11) | 30 | 4.45 | (2.89 | ,6.83) | 40 | 3.90 | (2.64 | ,5.74) |
|  | Hypertension+MS | 9,076 | (1.8) | 1,417 | 1.39 | (1.30 | ,1.48) | 334 | 2.68 | (2.32 | ,3.10) | 98 | 3.86 | (2.94 | ,5.08) | 109 | 2.71 | (2.10 | ,3.49) |
|  | Hypertension+Hyperlipidemia | 24,141 | (4.7) | 1,981 | 1.18 | (1.12 | ,1.25) | 492 | 2.49 | (2.20 | ,2.83) | 140 | 3.20 | (2.50 | ,4.09) | 156 | 2.42 | (1.93 | ,3.03) |
|  | CKD+Diabetes | 534 | (0.1) | 236 | 3.51 | (3.03 | ,4.07) | 33 | 3.48 | (2.27 | ,5.33) | 13 | 5.90 | (3.00 | ,11.61) | 8 | 1.94 | (0.72 | ,5.22) |
|  | CKD+MS | 67 | (0.0) | 14 | 1.26 | (0.68 | ,2.34) | 4 | 3.09 | (0.99 | ,9.60) | 1 | 4.53 | (0.63 | ,32.39) | 1 | 3.29 | (0.46 | ,23.47) |
|  | CKD+Hyperlipidemia | 3,456 | (0.7) | 575 | 1.49 | (1.35 | ,1.63) | 103 | 2.14 | (1.69 | ,2.70) | 29 | 2.29 | (1.44 | ,3.63) | 29 | 1.92 | (1.24 | ,2.97) |
|  | Diabetes+MS | 321 | (0.1) | 63 | 1.99 | (1.53 | ,2.58) | 11 | 2.29 | (1.14 | ,4.60) | 3 | 2.55 | (0.63 | ,10.30) | 2 | 1.78 | (0.44 | ,7.19) |
|  | Diabetes+Hyperlipidemia | 1,180 | (0.2) | 204 | 1.99 | (1.70 | ,2.32) | 41 | 3.48 | (2.47 | ,4.92) | 15 | 5.15 | (2.85 | ,9.29) | 18 | 5.43 | (3.30 | ,8.92) |
|  | MS+Hyperlipidemia | 5,391 | (1.0) | 369 | 1.10 | (0.98 | ,1.23) | 50 | 1.33 | (0.97 | ,1.81) | 13 | 1.24 | (0.63 | ,2.43) | 12 | 0.89 | (0.46 | ,1.73) |
| Three components | Hypertension+CKD+Diabetes | 701 | (0.1) | 365 | 3.17 | (2.82 | ,3.57) | 70 | 4.92 | (3.74 | ,6.47) | 18 | 5.36 | (3.13 | ,9.19) | 23 | 4.25 | (2.54 | ,7.11) |
|  | Hypertension+CKD+MS | 2,243 | (0.4) | 915 | 1.99 | (1.84 | ,2.15) | 250 | 4.02 | (3.41 | ,4.74) | 59 | 3.88 | (2.73 | ,5.52) | 83 | 3.89 | (2.90 | ,5.22) |
|  | Hypertension+CKD+Hyperlipidemia | 3,558 | (0.7) | 1,074 | 1.83 | (1.71 | ,1.97) | 296 | 4.06 | (3.48 | ,4.73) | 69 | 3.96 | (2.88 | ,5.44) | 104 | 4.35 | (3.34 | ,5.67) |
|  | Hypertension+Diabetes+MS | 3,040 | (0.6) | 790 | 2.16 | (1.99 | ,2.34) | 194 | 4.33 | (3.63 | ,5.17) | 54 | 5.13 | (3.62 | ,7.28) | 65 | 4.53 | (3.34 | ,6.14) |
|  | Hypertension+MS+Hyperlipidemia | 27,362 | (5.3) | 3,419 | 1.32 | (1.26 | ,1.38) | 881 | 2.87 | (2.58 | ,3.21) | 241 | 3.42 | (2.75 | ,4.26) | 291 | 2.96 | (2.44 | ,3.59) |
|  | Hypertension+Diabetes+Hyperlipidemia | 377 | (0.07) | 73 | 2.37 | (1.85 | ,3.03) | 16 | 4.19 | (2.42 | ,7.26) | 3 | 2.72 | (0.67 | ,11.00) | 7 | 5.88 | (2.60 | ,13.26) |
|  | CKD+Diabetes+MS | 67 | (0.0) | 34 | 3.37 | (2.37 | ,4.80) | 3 | 2.55 | (0.82 | ,7.94) | 3 | 10.73 | (3.41 | ,33.76) | 0 | 0.00 | (0.00 | . |
|  | CKD+Diabetes+Hyperlipidemia | 328 | (0.1) | 128 | 2.91 | (2.40 | ,3.54) | 22 | 4.05 | (2.50 | ,6.56) | 15 | 11.06 | (5.97 | ,20.47) | 3 | 2.19 | (0.70 | ,6.84) |
|  | CKD+MS+Hyperlipidemia | 647 | (0.1) | 139 | 1.33 | (1.10 | ,1.61) | 26 | 2.05 | (1.33 | ,3.17) | 7 | 2.53 | (1.12 | ,5.74) | 9 | 1.85 | (0.82 | ,4.17) |
|  | Diabetes+MS+Hyperlipidemia | 1,724 | (0.3) | 342 | 2.05 | (1.82 | ,2.31) | 58 | 2.86 | (2.12 | ,3.85) | 21 | 4.45 | (2.69 | ,7.37) | 18 | 2.65 | (1.54 | ,4.55) |
| Four components | Hypertension+CKD+Diabetes+MS | 1,388 | (0.3) | 715 | 3.38 | (3.10 | ,3.69) | 180 | 6.93 | (5.78 | ,8.31) | 65 | 10.56 | (7.63 | ,14.60) | 54 | 6.60 | (4.77 | ,9.14) |
|  | CKD+Diabetes+MS+Hyperlipidemia | 545 | (0.1) | 227 | 3.06 | (2.64 | ,3.55) | 42 | 4.94 | (3.53 | ,6.93) | 21 | 11.15 | (6.88 | ,18.07) | 13 | 4.69 | (2.55 | ,8.62) |
|  | Hypertension+Diabetes+MS+Hyperlipidemia | 6,830 | (1.3) | 1,697 | 2.08 | (1.96 | ,2.21) | 371 | 3.83 | (3.33 | ,4.40) | 124 | 5.70 | (4.40 | ,7.37) | 108 | 3.11 | (2.39 | ,4.04) |
|  | Hypertension+CKD+MS+Hyperlipidemia | 7,080 | (1.4) | 2,702 | 2.08 | (1.97 | ,2.19) | 820 | 5.06 | (4.51 | ,5.67) | 216 | 5.98 | (4.75 | ,7.52) | 252 | 4.90 | (3.99 | ,6.01) |
|  | Hypertension+CKD+Diabetes+Hyperlipidemia | 158 | (0.03) | 65 | 2.45 | (1.85 | ,3.25) | 16 | 5.71 | (3.36 | ,9.71) | 7 | 11.86 | (5.53 | ,25.42) | 5 | 4.95 | (1.84 | ,13.35) |
| Five components | Hypertension+CKD+Diabetes+MS+Hyperlipidemia | 4,517 | (0.9) | 2,317 | 3.71 | (3.52 | ,3.92) | 545 | 7.12 | (6.27 | ,8.09) | 187 | 11.04 | (8.71 | ,13.99) | 170 | 6.76 | (5.39 | ,8.48) |

Hazard ratios were adjusted for age, sex, educational levels, smoking status, drinking status, and physical activity.

Abbreviations: CKM: cardiovascular–kidney–metabolic syndrome; N: number of participants; CKD: chronic kidney disease; CVD: cardiovascular disease; HD: heart disease: DM: diabetes mellitus; MS: metabolic syndrome.

# Table S4-2. Hazard ratios for risks of cause-specific mortality and end-stage kidney disease stratified by number and type of cardiovascular–kidney–metabolic syndrome component

|  |  | DM mortality | | | | Kidney diseases mortality | | | | Expanded CVD mortality | | | | ESKD | | | |
| --- | --- | --- | --- | --- | --- | --- | --- | --- | --- | --- | --- | --- | --- | --- | --- | --- | --- |
|  |  | Deaths | HR | (95% CI) | |  | HR | (95% CI) | | Deaths | HR | (95% CI) | | ESKD | HR | (95% CI) | |
| Zero components |  | 62 | Ref. |  |  | 41 | Ref. |  |  | 864 | Ref. |  |  | 123 | Ref. |  |  |
| One component | Hypertension only | 114 | 2.39 | (1.65 | ,3.47) | 89 | 2.40 | (1.55 | ,3.70) | 1,821 | 2.43 | (2.22 | ,2.67) | 117 | 2.13 | (1.60 | ,2.84) |
|  | CKD only | 19 | 2.67 | (1.48 | ,4.82) | 35 | 5.96 | (3.51 | ,10.13) | 250 | 1.91 | (1.63 | ,2.25) | 141 | 16.35 | (12.51 | ,21.36) |
|  | Diabetes only | 69 | 38.85 | (25.94 | ,58.17) | 15 | 12.84 | (6.79 | ,24.30) | 156 | 4.95 | (4.08 | ,6.00) | 69 | 43.77 | (31.64 | ,60.56) |
|  | Metabolic syndrome only | 3 | 3.12 | (0.43 | ,22.67) | 1 | 3.86 | (0.53 | ,28.32) | 14 | 2.06 | (1.13 | ,3.73) | 4 | 6.15 | (1.52 | ,24.93) |
|  | Hyperlipidemia only | 33 | 1.89 | (1.14 | ,3.14) | 14 | 1.31 | (0.66 | ,2.62) | 341 | 1.39 | (1.21 | ,1.60) | 45 | 1.65 | (1.12 | ,2.42) |
| Two components | Hypertension+CKD | 64 | 5.29 | (3.50 | ,7.98) | 173 | 15.47 | (10.26 | ,23.33) | 1,153 | 4.72 | (4.25 | ,5.24) | 381 | 51.75 | (40.98 | ,65.35) |
|  | Hypertension+Diabetes | 67 | 43.32 | (29.04 | ,64.62) | 12 | 8.92 | (4.34 | ,18.33) | 179 | 5.71 | (4.76 | ,6.84) | 36 | 31.77 | (21.23 | ,47.55) |
|  | Hypertension+MS | 59 | 6.17 | (4.01 | ,9.51) | 21 | 3.51 | (1.98 | ,6.21) | 414 | 2.94 | (2.57 | ,3.35) | 26 | 4.14 | (2.62 | ,6.53) |
|  | Hypertension+Hyperlipidemia | 55 | 4.26 | (2.79 | ,6.51) | 15 | 1.81 | (0.96 | ,3.41) | 562 | 2.61 | (2.31 | ,2.94) | 45 | 2.69 | (1.80 | ,4.01) |
|  | CKD+Diabetes | 48 | 116.58 | (75.56 | ,179.89) | 12 | 32.25 | (15.23 | ,68.29) | 93 | 10.75 | (8.42 | ,13.73) | 45 | 135.66 | (91.08 | ,202.08) |
|  | CKD+MS | 1 | 15.91 | (2.19 | ,115.66) | 0 | 0.00 |  |  | 5 | 3.70 | (1.39 | ,9.89) | 3 | 22.77 | (3.17 | ,163.38) |
|  | CKD+Hyperlipidemia | 15 | 3.48 | (1.63 | ,7.42) | 14 | 5.57 | (2.71 | ,11.45) | 132 | 2.41 | (1.95 | ,2.98) | 67 | 25.45 | (18.11 | ,35.76) |
|  | Diabetes+MS | 11 | 48.06 | (24.71 | ,93.46) | 0 | 0.00 |  |  | 22 | 4.89 | (3.10 | ,7.72) | 9 | 43.91 | (21.32 | ,90.42) |
|  | Diabetes+Hyperlipidemia | 34 | 45.44 | (28.01 | ,73.73) | 7 | 13.17 | (5.47 | ,31.71) | 82 | 6.33 | (4.92 | ,8.14) | 35 | 56.02 | (37.33 | ,84.07) |
|  | MS+Hyperlipidemia | 16 | 6.55 | (3.58 | ,11.98) | 4 | 2.51 | (0.89 | ,7.14) | 70 | 1.70 | (1.31 | ,2.22) | 10 | 3.20 | (1.62 | ,6.33) |
| Three components | Hypertension+CKD+Diabetes | 75 | 95.09 | (63.35 | ,142.76) | 15 | 26.89 | (14.10 | ,51.28) | 160 | 10.54 | (8.70 | ,12.77) | 65 | 166.47 | (117.38 | ,236.08) |
|  | Hypertension+CKD+MS | 33 | 9.16 | (5.59 | ,15.01) | 49 | 15.92 | (9.84 | ,25.76) | 332 | 4.96 | (4.29 | ,5.74) | 97 | 57.23 | (42.46 | ,77.13) |
|  | Hypertension+CKD+Hyperlipidemia | 28 | 7.23 | (4.37 | ,11.98) | 57 | 17.14 | (10.77 | ,27.28) | 381 | 4.94 | (4.31 | ,5.66) | 167 | 67.57 | (51.93 | ,87.93) |
|  | Hypertension+Diabetes+MS | 121 | 45.87 | (31.93 | ,65.89) | 17 | 7.97 | (4.26 | ,14.88) | 332 | 6.89 | (5.98 | ,7.95) | 59 | 30.72 | (21.75 | ,43.40) |
|  | Hypertension+MS+Hyperlipidemia | 146 | 6.60 | (4.62 | ,9.43) | 50 | 2.92 | (1.81 | ,4.72) | 1,077 | 3.12 | (2.82 | ,3.45) | 97 | 5.41 | (4.04 | ,7.25) |
|  | Hypertension+Diabetes+Hyperlipidemia | 10 | 49.46 | (24.06 | ,101.67) | 1 | 6.80 | (0.93 | ,49.93) | 27 | 6.88 | (4.54 | ,10.43) | 10 | 58.74 | (30.58 | ,112.80) |
|  | CKD+Diabetes+MS | 5 | 74.67 | (29.46 | ,189.26) | 1 | 16.59 | (2.25 | ,122.06) | 9 | 7.09 | (3.67 | ,13.69) | 3 | 52.03 | (12.81 | ,211.32) |
|  | CKD+Diabetes+Hyperlipidemia | 23 | 84.14 | (49.29 | ,143.62) | 16 | 63.17 | (32.17 | ,124.06) | 61 | 10.94 | (8.18 | ,14.64) | 54 | 287.02 | (200.71 | ,410.46) |
|  | CKD+MS+Hyperlipidemia | 10 | 9.52 | (4.04 | ,22.43) | 10 | 17.42 | (8.23 | ,36.87) | 46 | 3.21 | (2.29 | ,4.49) | 17 | 33.40 | (19.36 | ,57.64) |
|  | Diabetes+MS+Hyperlipidemia | 56 | 49.24 | (32.78 | ,73.96) | 19 | 16.29 | (8.48 | ,31.32) | 133 | 6.20 | (5.07 | ,7.58) | 64 | 52.65 | (37.36 | ,74.20) |
| Four components | Hypertension+CKD+Diabetes+MS | 130 | 90.39 | (62.90 | ,129.88) | 38 | 28.30 | (16.86 | ,47.49) | 348 | 12.48 | (10.84 | ,14.37) | 143 | 181.94 | (137.84 | ,240.16) |
|  | CKD+Diabetes+MS+Hyperlipidemia | 50 | 95.27 | (61.62 | ,147.29) | 13 | 28.09 | (13.66 | ,57.79) | 105 | 10.98 | (8.76 | ,13.77) | 71 | 199.32 | (143.17 | ,277.50) |
|  | Hypertension+Diabetes+MS+Hyperlipidemia | 292 | 47.09 | (33.82 | ,65.56) | 51 | 10.83 | (6.78 | ,17.31) | 714 | 6.63 | (5.92 | ,7.42) | 183 | 42.46 | (32.82 | ,54.93) |
|  | Hypertension+CKD+MS+Hyperlipidemia | 133 | 14.21 | (9.90 | ,20.41) | 177 | 21.52 | (14.34 | ,32.30) | 1,130 | 6.45 | (5.81 | ,7.15) | 428 | 79.99 | (63.42 | ,100.90) |
|  | Hypertension+CKD+Diabetes+Hyperlipidemia | 15 | 66.02 | (32.02 | ,136.12) | 4 | 9.04 | (1.23 | ,66.50) | 35 | 9.19 | (6.11 | ,13.83) | 22 | 196.83 | (113.82 | ,340.38) |
| Five components | Hypertension+CKD+Diabetes+MS+Hyperlipidemia | 537 | 123.35 | (89.36 | ,170.26) | 179 | 45.62 | (30.37 | ,68.54) | 1,261 | 15.25 | (13.78 | ,16.88) | 698 | 270.95 | (216.69 | ,338.78) |

Hazard ratios were adjusted for age, sex, educational levels, smoking status, drinking status, and physical activity.

Abbreviations: CKM: cardiovascular–kidney–metabolic syndrome; N: number of participants; CKD: chronic kidney disease; CVD: cardiovascular disease; HD: heart disease: DM: diabetes mellitus; MS: metabolic syndrome; ESKD: end-stage kidney disease; Ref: reference group.
