# Supplementary material for: Cardiovascular–kidney–metabolic syndrome and all-cause and cardiovascular mortality: A retrospective cohort study
Source: PLoS Med. 2025 Jun 26;22(6):e1004629. doi: 10.1371/journal.pmed.1004629 (PMC12200875; doi:10.1371/journal.pmed.1004629)
Supplement: S5 Table — (DOCX) [file pmed.1004629.s005.docx]

# Table S5. Remaining years of life stratified by CKM stage and number of components for male and female

|  | Male |  |  |  |  |  |  |  |  |  |  |  |  |  |  |  |  |  |  |  |  |
| --- | --- | --- | --- | --- | --- | --- | --- | --- | --- | --- | --- | --- | --- | --- | --- | --- | --- | --- | --- | --- | --- |
|  | CKM  Stage 0 | CKM  Stage 1 | Difference |  |  | CKM  Stage 2 | Difference |  |  | CKM  Stage 3 | Difference |  |  | CKM  Stage 4 | Difference |  |  |  |  |  |  |
| 30 | 54.2 | 54.0 | 0.2 | (-0.03 | ,0.39) | 51.7 | 2.5 | (2.36 | ,2.55) | 48.0 | 6.2 | (5.03 | ,7.38) | 47.2 | 7.0 | (6.42 | ,7.58) |  |  |  |  |
| 40 | 44.4 | 44.3 | 0.2 | (-0.03 | ,0.38) | 42.1 | 2.3 | (2.25 | ,2.44) | 38.0 | 6.5 | (5.29 | ,7.63) | 37.8 | 6.6 | (6.12 | ,7.10) |  |  |  |  |
| 50 | 34.9 | 34.6 | 0.2 | (0.01 | ,0.41) | 32.7 | 2.1 | (2.05 | ,2.23) | 28.4 | 6.5 | (5.58 | ,7.37) | 29.1 | 5.7 | (5.36 | ,6.13) |  |  |  |  |
| 60 | 25.6 | 25.4 | 0.2 | (0.00 | ,0.39) | 23.8 | 1.8 | (1.71 | ,1.87) | 20.6 | 5.0 | (4.51 | ,5.40) | 20.9 | 4.6 | (4.35 | ,4.90) |  |  |  |  |
| 70 | 16.8 | 16.6 | 0.3 | (0.08 | ,0.44) | 15.5 | 1.3 | (1.22 | ,1.36) | 14.3 | 2.6 | (2.37 | ,2.76) | 13.6 | 3.3 | (3.08 | ,3.47) |  |  |  |  |
|  | 0 components | 1 component | |  |  | 2 components | |  |  | 3 components | |  |  | 4 components | |  |  | 5 components | |  |  |
| 30 | 54.5 | 53.0 | 1.5 | (1.36 | ,1.62) | 50.8 | 3.7 | (3.55 | ,3.93) | 50.0 | 4.5 | (4.24 | ,4.77) | 46.5 | 8.0 | (7.37 | ,8.68) | 40.7 | 13.8 | (12.07 | ,15.45) |
| 40 | 44.8 | 43.3 | 1.4 | (1.32 | ,1.57) | 41.2 | 3.6 | (3.40 | ,3.74) | 40.5 | 4.3 | (4.03 | ,4.48) | 37.5 | 7.3 | (6.88 | ,7.78) | 32.0 | 12.7 | (11.74 | ,13.73) |
| 50 | 35.2 | 33.8 | 1.4 | (1.25 | ,1.49) | 32.0 | 3.2 | (3.08 | ,3.40) | 31.4 | 3.8 | (3.58 | ,3.99) | 29.2 | 6.0 | (5.66 | ,6.32) | 24.3 | 10.9 | (10.20 | ,11.58) |
| 60 | 25.9 | 24.7 | 1.2 | (1.12 | ,1.35) | 23.1 | 2.8 | (2.67 | ,2.95) | 22.7 | 3.2 | (3.00 | ,3.36) | 20.9 | 5.1 | (4.81 | ,5.34) | 17.6 | 8.3 | (7.88 | ,8.79) |
| 70 | 17.2 | 16.2 | 1.0 | (0.94 | ,1.14) | 15.0 | 2.2 | (2.10 | ,2.33) | 14.7 | 2.5 | (2.36 | ,2.66) | 13.7 | 3.6 | (3.39 | ,3.79) | 11.2 | 6.0 | (5.71 | ,6.35) |
|  | Female |  |  |  |  |  |  |  |  |  |  |  |  |  |  |  |  |  |  |  |  |
|  | CKM  Stage 0 | CKM  Stage 1 | Difference |  |  | CKM  Stage 2 | Difference |  |  | CKM  Stage 3 | Difference |  |  | CKM  Stage 4 | Difference |  |  |  |  |  |  |
| 30 | 59.9 | 58.9 | 0.9 | (0.68 | ,1.13) | 55.8 | 4.1 | (3.98 | ,4.19) | 52.8 | 7.0 | (5.41 | ,8.62) | 50.9 | 8.9 | (8.38 | ,9.44) |  |  |  |  |
| 40 | 50.0 | 49.5 | 0.5 | (0.30 | ,0.74) | 46.0 | 4.0 | (3.93 | ,4.12) | 42.8 | 7.2 | (5.59 | ,8.80) | 41.5 | 8.5 | (8.07 | ,8.92) |  |  |  |  |
| 50 | 40.4 | 40.3 | 0.1 | (-0.13 | ,0.29) | 36.5 | 3.9 | (3.78 | ,3.95) | 32.8 | 7.5 | (5.91 | ,9.12) | 32.2 | 8.2 | (7.84 | ,8.53) |  |  |  |  |
| 60 | 30.9 | 31.2 | -0.3 | (-0.51 | ,-0.10) | 27.2 | 3.6 | (3.55 | ,3.70) | 24.2 | 6.6 | (5.90 | ,7.36) | 23.7 | 7.2 | (6.95 | ,7.43) |  |  |  |  |
| 70 | 21.6 | 22.1 | -0.5 | (-0.66 | ,-0.27) | 18.5 | 3.2 | (3.09 | ,3.22) | 16.9 | 4.7 | (4.32 | ,5.03) | 15.7 | 6.0 | (5.78 | ,6.13) |  |  |  |  |
|  | 0 components | 1 component | |  |  | 2 components | |  |  | 3 components | |  |  | 4 components | |  |  | 5 components | |  |  |
| 30 | 60.5 | 57.6 | 2.9 | (2.73 | ,3.01) | 55.0 | 5.5 | (5.19 | ,5.75) | 53.6 | 6.9 | (6.38 | ,7.45) | 50.6 | 9.9 | (8.89 | ,10.87) | 44.2 | 16.3 | (14.61 | ,18.00) |
| 40 | 50.7 | 47.8 | 2.9 | (2.73 | ,3.00) | 45.5 | 5.2 | (5.03 | ,5.45) | 44.2 | 6.5 | (6.14 | ,6.80) | 41.4 | 9.3 | (8.66 | ,9.87) | 34.9 | 15.8 | (14.73 | ,16.92) |
| 50 | 41.0 | 38.3 | 2.7 | (2.62 | ,2.87) | 36.0 | 5.1 | (4.90 | ,5.25) | 35.0 | 6.0 | (5.79 | ,6.25) | 32.3 | 8.8 | (8.38 | ,9.15) | 25.8 | 15.3 | (14.47 | ,16.08) |
| 60 | 31.6 | 29.0 | 2.6 | (2.53 | ,2.75) | 26.8 | 4.8 | (4.70 | ,4.98) | 26.1 | 5.5 | (5.36 | ,5.69) | 23.7 | 7.9 | (7.68 | ,8.17) | 18.4 | 13.2 | (12.78 | ,13.71) |
| 70 | 22.5 | 20.1 | 2.4 | (2.32 | ,2.52) | 18.1 | 4.4 | (4.24 | ,4.47) | 17.4 | 5.1 | (4.97 | ,5.23) | 15.5 | 7.0 | (6.79 | ,7.15) | 11.9 | 10.6 | (10.24 | ,10.86) |

Difference: It represents the variation in remaining life expectancy across different CKM stages or components, with CKM stage 0 or 0 components used as the reference group.

In this table, life expectancy refers to the average expected remaining years of life for individuals at ages 30, 40, 50, 60, and 70 years

Abbreviations: CKM: cardiovascular–kidney–metabolic syndrome.
