# Supplementary material for: Cardiovascular–kidney–metabolic syndrome and all-cause and cardiovascular mortality: A retrospective cohort study
Source: PLoS Med. 2025 Jun 26;22(6):e1004629. doi: 10.1371/journal.pmed.1004629 (PMC12200875; doi:10.1371/journal.pmed.1004629)
Supplement: S6 Table — (DOCX) [file pmed.1004629.s006.docx]

# Table S6. Prevalence of CKM components in study cohort stratified by age

|  |  | Total |  | Age < 65 years | | Age ≥ 65 years | |
| --- | --- | --- | --- | --- | --- | --- | --- |
|  |  | N | (%) | N | (%) | N | (%) |
| 0 components |  | 302,667 | (58.7) | 297,931 | (61.5) | 4,736 | (15.0) |
| Any CKM components | Hypertension | 185,881 | (36.1) | 161,358 | (33.3) | 24,523 | (77.9) |
|  | CKD | 49,637 | (9.6) | 36,951 | (7.6) | 12,686 | (40.3) |
|  | Diabetes | 26,679 | (5.2) | 20,647 | (4.3) | 6,032 | (19.2) |
|  | Metabolic syndrome | 70,907 | (13.8) | 58,448 | (12.1) | 12,459 | (39.6) |
|  | Hyperlipidemia | 133,746 | (25.9) | 121,279 | (25.1) | 12,467 | (39.6) |
|  | Two or more | 117,212 | (22.7)* | 97,367 | (20.1) | 19,845 | (63.1) |
|  | Three or more | 60,565 | (11.7)** | 48,346 | (10.0) | 12,219 | (38.8) |
|  | Four or more | 20,518 | (4.0) | 14,419 | (3.0) | 6,099 | (19.4) |
|  | Two or more (among any CKM components) | 117,212 | (44.4)* | 97,367 | (41.3) | 19,845 | (69.9) |
|  | Three or more (among any CKM components) | 60,565 | (22.9)** | 48,346 | (20.5) | 12,219 | (43.0) |
|  | Four or more (among any CKM components) | 20,518 | (7.8) | 14,419 | (6.1) | 6,099 | (21.5) |

Abbreviations: CKM: cardiovascular–kidney–metabolic syndrome; CKD: chronic kidney disease; Ref: reference; N: number of participants.

Number of total participants: 515,602; Number of any CKM participants: 264,038;

Number of total participants and age < 65 years: 484,130; Number of any CKM participants and age < 65 years: 235,637;

Number of total participants and age ≥ 65 years: 31,472; Number of any CKM participants and age ≥ 65 years: 28,401.

*One-fifth (22.7%= 117,212/515,602) of the participants had two or more CKM components. However, among those with any CKM component (N=264,038), two-fifths (44.4%= 117,212/264,038) had two or more components.

**One-ninth (11.7%= 60,565/515,602) of participants in the entire cohort had three or more CKM components. However, among those with any CKM component, the proportion with three or more components increased to one-fifths (22.9%= 60,565/264,038).
