# Supplementary material for: Cardiovascular–kidney–metabolic syndrome and all-cause and cardiovascular mortality: A retrospective cohort study
Source: PLoS Med. 2025 Jun 26;22(6):e1004629. doi: 10.1371/journal.pmed.1004629 (PMC12200875; doi:10.1371/journal.pmed.1004629)
Supplement: S7 Table — (DOCX) [file pmed.1004629.s007.docx]

# Table S7. Prevalence of self-reported medication and hazard ratios with risk of all-cause mortality by CKM components

|  |  | N | (%) | (%) among diseased | n of  deaths | HR | (95% CI) | |
| --- | --- | --- | --- | --- | --- | --- | --- | --- |
| Non-hypertension |  | 329,721 | (63.95) |  | 12,906 | REF |  |  |
| Hypertension | Screened hypertension (130/80) | 144,822 | (28.09) | (77.91) | 17,380 | 1.23 | (1.20 | ,1.27) |
|  | History or on medication | 41,059 | (7.96) | (22.09) | 11,303 | 1.62 | (1.57 | ,1.67) |
| Non-hypertension |  | 420,896 | (81.63) |  | 19,957 | REF |  |  |
| Hypertension | Screened hypertension (140/90) | 53,647 | (10.40) | (56.65) | 10,329 | 1.28 | (1.25 | ,1.32) |
|  | History or on medication | 41,059 | (7.96) | (43.35) | 11,303 | 1.56 | (1.52 | ,1.60) |
| Non-CKD |  | 465,965 | (90.37) |  | 27,265 | REF |  |  |
| CKD | Screened CKD | 47,990 | (9.31) | (96.68) | 13,712 | 1.62 | (1.58 | ,1.66) |
|  | History or on medication | 1,647 | (0.32) | (3.32) | 612 | 2.51 | (2.30 | ,2.73) |
| Non-DM |  | 488,923 | (94.83) |  | 33,403 | REF |  |  |
| DM | Screened DM | 12,123 | (2.35) | (45.44) | 2,911 | 1.56 | (1.49 | ,1.63) |
|  | History or on medication | 14,556 | (2.82) | (54.56) | 5,275 | 2.11 | (2.04 | ,2.17) |
| Non-Metabolic syndrome |  | 444,695 | (86.25) |  | 26,376 | REF |  |  |
| Metabolic syndrome | Screened Metabolic syndrome | 43,719 | (8.48) | (61.66) | 6,538 | 1.13 | (1.10 | ,1.17) |
|  | History or on medication  (hypertension, DM or hypertriglyceride) | 27,188 | (5.27) | (38.34) | 8,675 | 1.67 | (1.62 | ,1.71) |
| Non-hyertriglyceride |  | 381,856 | (74.06) |  | 24,340 | REF |  |  |
| Hypertriglyceride | Screened hypertriglyceride | 129,859 | (25.19) | (97.09) | 16,395 | 1.15 | (1.12 | ,1.17) |
|  | History or on medication | 3,887 | (0.75) | (2.91) | 854 | 1.24 | (1.16 | ,1.34) |

Abbreviations: CKM: cardiovascular–kidney–metabolic syndrome; N: number of participants; CKD: chronic kidney disease; DM: diabetes mellitus; HR: hazard ratio; CI: confidence interval; REF: reference group
