# Supplementary material for: Cardiovascular–kidney–metabolic syndrome and all-cause and cardiovascular mortality: A retrospective cohort study
Source: PLoS Med. 2025 Jun 26;22(6):e1004629. doi: 10.1371/journal.pmed.1004629 (PMC12200875; doi:10.1371/journal.pmed.1004629)
Supplement: S8 Table — (DOCX) [file pmed.1004629.s008.docx]

# Table S8. Prevalence of cardiovascular–kidney–metabolic syndrome in this cohort and in the US National Health and Nutrition Examination Survey

|  |  | Prevalence in this cohort | (%) among diseased |  | Prevalence in US NHANES | (%) among diseased |
| --- | --- | --- | --- | --- | --- | --- |
| Age 20–44 years | Stage 0 | 37.8 |  |  | 17.4 |  |
|  | Stage 1 | 22.0 | (35.3) |  | 37.6 | (45.5) |
|  | Stage 2 | 39.0 | (62.6) |  | 43.2 | (52.2) |
|  | Stage 3 | 0.0 | (0.0) |  | 0.2 | (0.2) |
|  | Stage 4 | 1.3 | (2.0) |  | 1.7 | (2.1) |
| Age 45–64 years | Stage 0 | 10.8 |  |  | 5.5 |  |
|  | Stage 1 | 16.5 | (18.5) |  | 21.0 | (22.2) |
|  | Stage 2 | 64.5 | (72.3) |  | 63.8 | (67.5) |
|  | Stage 3 | 1.6 | (1.8) |  | 1.2 | (1.2) |
|  | Stage 4 | 6.5 | (7.3) |  | 8.6 | (9.1) |
| Age ≥ 65 years | Stage 0 | 3.1 |  |  | 1.8 |  |
|  | Stage 1 | 5.3 | (5.5) |  | 8.4 | (8.6) |
|  | Stage 2 | 47.8 | (49.3) |  | 39.0 | (39.7) |
|  | Stage 3 | 24.3 | (25.1) |  | 24.6 | (25.1) |
|  | Stage 4 | 19.4 | (20.1) |  | 26.2 | (26.6) |
| Overall | Stage 0 | 28.5 |  |  | 10.1 |  |
|  | Stage 1 | 19.5 | (27.3) |  | 26.0 | (28.9) |
|  | Stage 2 | 46.3 | (64.7) |  | 49.7 | (55.3) |
|  | Stage 3 | 1.9 | (2.7) |  | 5.3 | (5.9) |
|  | Stage 4 | 3.8 | (5.3) |  | 8.9 | (9.9) |

Prevalence for the US National Health and Nutrition Examination Survey was from Minhas AMK et al.[1].

**Reference**

1. Minhas AMK, Mathew RO, Sperling LS, Nambi V, Virani SS, Navaneethan SD, et al. Prevalence of the Cardiovascular-Kidney-Metabolic Syndrome in the United States. J Am Coll Cardiol. 2024. Epub 20240401. doi: 10.1016/j.jacc.2024.03.368. PubMed PMID: 38583160.
