# Supplementary material for: Cardiovascular–kidney–metabolic syndrome and all-cause and cardiovascular mortality: A retrospective cohort study
Source: PLoS Med. 2025 Jun 26;22(6):e1004629. doi: 10.1371/journal.pmed.1004629 (PMC12200875; doi:10.1371/journal.pmed.1004629)
Supplement: S9 Table — (DOCX) [file pmed.1004629.s009.docx]

# Table S9. Population attributable fractions of cardiovascular–kidney–metabolic syndrome stages and components for all-cause and cardiovascular-disease mortality

|  |  | All-cause mortality | |  |  |  |  |  |  |  | CVD mortality |  |  |  |  |  |  |  |  |
| --- | --- | --- | --- | --- | --- | --- | --- | --- | --- | --- | --- | --- | --- | --- | --- | --- | --- | --- | --- |
|  | Prevalence (%) | Hazard  ratio | (95% CI) | | PAF (%) | (95% CI) | | PAF* (%) | (95% CI) | | Hazard  ratio | (95% CI) | | PAF (%) | (95% CI) | | PAF* (%) | (95% CI) | |
| CKM stage 1 | 19.5 | 0.96 | (0.91 | ,1.02) | -0.7 | (-1.76 | ,0.32) | -0.6 | (-1.48 | ,0.24) | 1.13 | (0.95 | ,1.35) | 2.5 | (-0.99 | ,6.48) | 1.2 | (-0.51 | ,2.71) |
| CKM stage 2 | 46.3 | 1.36 | (1.30 | ,1.42) | 14.1 | (12.08 | ,16.24) | 13.4 | (11.62 | ,15.12) | 2.89 | (2.51 | ,3.32) | 46.6 | (41.07 | ,51.84) | 39.3 | (35.69 | ,42.44) |
| CKM stage 3 | 1.9 | 2.13 | (2.02 | ,2.25) | 2.1 | (1.66 | ,2.68) | 1.8 | (1.65 | ,1.89) | 5.27 | (4.51 | ,6.16) | 7.6 | (5.49 | ,10.17) | 3.7 | (3.45 | ,3.93) |
| CKM stage 4 | 3.8 | 2.37 | (2.25 | ,2.49) | 4.9 | (4.18 | ,5.68) | 4.2 | (3.96 | ,4.41) | 7.42 | (6.40 | ,8.60) | 19.5 | (15.88 | ,23.47) | 10.9 | (10.37 | ,11.33) |
| CKM all |  |  |  |  |  |  |  | 18.74 | (15.75 | ,21.66) |  |  |  |  |  |  | 55.01 | (49.00 | ,60.41) |
| Hypertension | 36.1 | 1.35 | (1.32 | ,1.39) | 11.3 | (10.26 | ,12.25) |  |  |  | 2.35 | (2.21 | ,2.50) | 32.8 | (30.26 | ,35.22) |  |  |  |
| CKD | 9.6 | 1.65 | (1.61 | ,1.69) | 5.9 | (5.40 | ,6.37) |  |  |  | 1.85 | (1.76 | ,1.95) | 7.6 | (6.64 | ,8.56) |  |  |  |
| Diabetes | 5.2 | 1.89 | (1.84 | ,1.95) | 4.4 | (3.97 | ,4.89) |  |  |  | 1.83 | (1.73 | ,1.94) | 4.1 | (3.45 | ,4.88) |  |  |  |
| Metabolic syndrome | 13.8 | 1.40 | (1.37 | ,1.43) | 5.2 | (4.70 | ,5.66) |  |  |  | 1.72 | (1.64 | ,1.81) | 9.1 | (7.99 | ,10.17) |  |  |  |
| Hyper triglyceride | 25.9 | 1.15 | (1.13 | ,1.18) | 3.7 | (3.11 | ,4.38) |  |  |  | 1.40 | (1.34 | ,1.47) | 9.4 | (7.93 | ,10.85) |  |  |  |
|  |  |  |  |  |  |  |  |  |  |  |  |  |  |  |  |  |  |  |  |
| One component | 28.5 | 1.21 | (1.17 | ,1.26) | 5.7 | (4.63 | ,6.82) |  |  |  | 2.02 | (1.84 | ,2.22) | 22.6 | (19.25 | ,25.99) |  |  |  |
| Two components | 11.0 | 1.49 | (1.43 | ,1.54) | 5.1 | (4.44 | ,5.74) |  |  |  | 2.84 | (2.58 | ,3.13) | 16.8 | (14.49 | ,19.29) |  |  |  |
| Three components | 7.8 | 1.57 | (1.51 | ,1.63) | 4.2 | (3.67 | ,4.79) |  |  |  | 3.17 | (2.88 | ,3.50) | 14.4 | (12.37 | ,16.71) |  |  |  |
| Four components | 3.1 | 2.12 | (2.03 | ,2.21) | 3.4 | (2.84 | ,3.92) |  |  |  | 4.54 | (4.10 | ,5.02) | 9.9 | (8.07 | ,11.95) |  |  |  |
| Five components | 0.9 | 3.53 | (3.34 | ,3.72) | 2.2 | (1.39 | ,3.03) |  |  |  | 6.68 | (5.88 | ,7.58) | 4.7 | (2.87 | ,7.02) |  |  |  |

Abbreviations: CKM: cardiovascular–kidney–metabolic syndrome; CI: confidence interval; PAF: population attributable fraction; CKD: chronic kidney disease.

*The total PAF of CKM was estimated by the following formula

$\sum_{i=1}^{n} Pi(RRi-1)$ / $\sum_{i=1}^{n} Pi(RRi-1)+1$

where *i* signifies the stages of CKM (i = 1,…, n).
