# Supplementary material for: Cardiovascular–kidney–metabolic syndrome and all-cause and cardiovascular mortality: A retrospective cohort study
Source: PLoS Med. 2025 Jun 26;22(6):e1004629. doi: 10.1371/journal.pmed.1004629 (PMC12200875; doi:10.1371/journal.pmed.1004629)
Supplement: S10 Table — (DOCX) [file pmed.1004629.s010.docx]

# Table S10. Age group–specific and sex-specific risks of all-cause mortality stratified by cardiovascular–kidney–metabolic syndrome status

|  | Total cohort | | | | | Male | | | | | Female | | | | | Age < 65 yr | | | | | Age ≥ 65 yr | | | | |
| --- | --- | --- | --- | --- | --- | --- | --- | --- | --- | --- | --- | --- | --- | --- | --- | --- | --- | --- | --- | --- | --- | --- | --- | --- | --- |
|  | All-cause mortality | | | | | All-cause mortality | | | | | All-cause mortality | | | | | All-cause mortality | | | | | All-cause mortality | | | | |
| CKM | N | N of  deaths | HR* | (95% CI) | | N | N of  deaths | HR* | (95% CI) | | N | N of  deaths | HR* | (95% CI) | | N | N of  deaths | HR* | (95% CI) | | N | N of  deaths | HR* | (95% CI) | |
| Stage 0 | 147,024 | 3,002 | 1.00 |  |  | 41,626 | 1,493 | Ref. |  |  | 105,398 | 1,509 | Ref. |  |  | 146,063 | 2,553 | Ref. |  |  | 961 | 449 | Ref. |  |  |
| Stage 1 | 100,585 | 3,280 | 0.96 | (0.91 | ,1.02) | 52,856 | 1,861 | 0.94 | (0.87 | ,1.02) | 47,729 | 1,419 | 0.98 | (0.90 | ,1.06) | 98,910 | 2,688 | 0.98 | (0.93 | ,1.04) | 1,675 | 592 | 0.83 | (0.72 | ,0.96) |
| Stage 2 | 238,647 | 22,469 | 1.36 | (1.30 | ,1.42) | 147,330 | 12,773 | 1.31 | (1.23 | ,1.39) | 91,317 | 9,696 | 1.41 | (1.32 | ,1.51) | 223,592 | 15,597 | 1.41 | (1.34 | ,1.48) | 15,055 | 6,872 | 1.02 | (0.92 | ,1.14) |
| Stage 3 | 9,925 | 6,415 | 2.13 | (2.02 | ,2.25) | 6,941 | 4,521 | 1.91 | (1.78 | ,2.04) | 2,984 | 1,894 | 2.65 | (2.42 | ,2.90) | 2,263 | 1,011 | 3.14 | (2.89 | ,3.41) | 7,662 | 5,404 | 1.49 | (1.33 | ,1.66) |
| Stage 4 | 19,421 | 6,423 | 2.37 | (2.25 | ,2.49) | 9,314 | 3,444 | 2.25 | (2.09 | ,2.41) | 10,107 | 2,979 | 2.50 | (2.31 | ,2.71) | 13,302 | 2,497 | 2.51 | (2.36 | ,2.68) | 6,119 | 3,926 | 1.71 | (1.53 | ,1.92) |
| All CKM† | 368,578 | 38,587 | 1.33 | (1.28 | ,1.39) | 216,441 | 22,599 | 1.33 | (1.26 | ,1.41) | 152,137 | 15,988 | 1.31 | (1.23 | ,1.40) | 338,067 | 21,793 | 1.37 | (1.31 | ,1.44) | 30,511 | 16,794 | 1.25 | (1.12 | ,1.39) |
|  |  |  |  |  |  |  |  |  |  |  |  |  |  |  |  |  |  |  |  |  |  |  |  |  |  |
| Zero components | 251,564 | 6,750 | 1.00 |  |  | 96,012 | 3,650 | Ref. |  |  | 155,552 | 3,100 | Ref. |  |  | 248,493 | 5,455 | Ref. |  |  | 3,071 | 1,295 | Ref. |  |  |
| One component | 146,826 | 11,065 | 1.21 | (1.17 | ,1.26) | 88,515 | 6,788 | 1.21 | (1.15 | ,1.26) | 58,311 | 4,277 | 1.24 | (1.17 | ,1.31) | 138,270 | 6,839 | 1.21 | (1.16 | ,1.26) | 8,556 | 4,226 | 1.19 | (1.11 | ,1.28) |
| Two components | 56,647 | 8,772 | 1.49 | (1.43 | ,1.54) | 36,955 | 5,561 | 1.49 | (1.42 | ,1.56) | 19,692 | 3,211 | 1.49 | (1.40 | ,1.58) | 49,021 | 4,454 | 1.50 | (1.44 | ,1.57) | 7,626 | 4,318 | 1.39 | (1.30 | ,1.49) |
| Three components | 40,047 | 7,279 | 1.57 | (1.51 | ,1.63) | 25,027 | 4,024 | 1.55 | (1.48 | ,1.63) | 15,020 | 3,255 | 1.61 | (1.52 | ,1.71) | 33,927 | 3,976 | 1.69 | (1.61 | ,1.77) | 6,120 | 3,303 | 1.36 | (1.27 | ,1.46) |
| Four components | 16,001 | 5,406 | 2.12 | (2.03 | ,2.21) | 9,086 | 2,892 | 2.00 | (1.90 | ,2.12) | 6,915 | 2,514 | 2.33 | (2.18 | ,2.49) | 11,505 | 2,498 | 2.48 | (2.35 | ,2.62) | 4,496 | 2,908 | 1.75 | (1.62 | ,1.88) |
| Five components | 4,517 | 2,317 | 3.53 | (3.34 | ,3.72) | 2,472 | 1,177 | 3.10 | (2.88 | ,3.33) | 2,045 | 1,140 | 4.24 | (3.90 | ,4.60) | 2,914 | 1,124 | 4.74 | (4.41 | ,5.10) | 1,603 | 1,193 | 2.60 | (2.38 | ,2.84) |
| Increase by one component |  |  | 1.22 | (1.21 | ,1.23) |  |  | 1.20 | (1.19 | ,1.21) |  |  | 1.26 | (1.24 | ,1.28) |  |  | 1.27 | (1.26 | ,1.29) |  |  | 1.16 | (1.14 | ,1.17) |

*Hazard ratios were adjusted for age, sex, educational level, smoking status, drinking status, and physical activity.

†All CKM does not include stage 0.

Abbreviations: CKM: cardiovascular–kidney–metabolic syndrome; N: number of participants; HR: hazard ratio; Ref: reference group.
