# Supplementary material for: Cardiovascular–kidney–metabolic syndrome and all-cause and cardiovascular mortality: A retrospective cohort study
Source: PLoS Med. 2025 Jun 26;22(6):e1004629. doi: 10.1371/journal.pmed.1004629 (PMC12200875; doi:10.1371/journal.pmed.1004629)
Supplement: S11 Table — (DOCX) [file pmed.1004629.s011.docx]

# Table S11. The distribution of baseline cardiovascular–kidney-metabolic syndrome status and follow-up CKM status among participants with a second visit

|  | 2nd visit CKM status | | |  |  |  |  |  |  |  |  |  |
| --- | --- | --- | --- | --- | --- | --- | --- | --- | --- | --- | --- | --- |
|  | Stage 0 | | Stage 1 | | Stage 2 | | Stage 3 | | Stage 4 | | Total |  |
| Baseline CKM status | N | (%) | N | (%) | N | (%) | N | (%) | N | (%) | N | (%) |
| Stage 0 | 44,914 | (20.4) | 9,676 | (4.4) | 10,958 | (5.0) | 30 | (0.0) | 388 | (0.2) | 65,966 | (29.9) |
| Stage 1 | 6,276 | (2.8) | 23,331 | (10.6) | 14,454 | (6.6) | 47 | (0.0) | 409 | (0.2) | 44,517 | (20.2) |
| Stage 2 | 10,280 | (4.7) | 13,377 | (6.1) | 73,298 | (33.3) | 1,566 | (0.7) | 2,457 | (1.1) | 100,978 | (45.8) |
| Stage 3 | 8 | (0.0) | 10 | (0.0) | 625 | (0.3) | 1,682 | (0.8) | 335 | (0.2) | 2,660 | (1.2) |
| Stage 4 | 92 | (0.0) | 90 | (0.0) | 547 | (0.2) | 118 | (0.1) | 5,414 | (2.5) | 6,261 | (2.8) |
| Total | 61,570 | (27.9) | 46,484 | (21.1) | 99,882 | (45.3) | 3,443 | (1.6) | 9,003 | (4.1) | 220,382 | (100.0) |

Abbreviations: CKM: cardiovascular–kidney–metabolic syndrome; N: number of participants;

The average follow-up time between these two examinations was 2.5 years (mean (SD): 2.5 (2.4)).
