# Supplementary material for: Cardiovascular–kidney–metabolic syndrome and all-cause and cardiovascular mortality: A retrospective cohort study
Source: PLoS Med. 2025 Jun 26;22(6):e1004629. doi: 10.1371/journal.pmed.1004629 (PMC12200875; doi:10.1371/journal.pmed.1004629)
Supplement: S12 Table — (DOCX) [file pmed.1004629.s012.docx]

# Table S12. Hazard ratios with risk of all-cause mortality stratified by cardiovascular–kidney–metabolic syndrome status among participants with 2nd visit, Time-dependent results

|  |  | Time-dependent results* | | | | | | | | | | |  |  |  |  |  |  |  |
| --- | --- | --- | --- | --- | --- | --- | --- | --- | --- | --- | --- | --- | --- | --- | --- | --- | --- | --- | --- |
| CKM |  | N | | n of deaths | | HR | | (95% CI) | | | | |  |  |  |  |  |  |  |
| Stage 0 |  | 61,570 | | 1,086 | | Ref. | |  | |  | | |  |  |  |  |  |  |  |
| Stage 1 |  | 46,484 | | 1,189 | | 1.39 | | (0.86 | | ,2.25) | | |  |  |  |  |  |  |  |
| Stage 2 |  | 99,882 | | 6,667 | | 2.35 | | (1.61 | | ,3.41) | | |  |  |  |  |  |  |  |
| Stage 3 |  | 3,443 | | 1,852 | | 4.61 | | (1.66 | | ,12.80) | | |  |  |  |  |  |  |  |
| Stage 4 |  | 9,003 | | 2,292 | | 2.46 | | (0.76 | | ,7.96) | | |  |  |  |  |  |  |  |
|  |  | |  | |  | |  | |  | |  |  | | |  |  |  |  |  |

Hazard ratios were adjusted for age, sex, educational level, smoking status, drinking status, and physical activity.

*Time-varying CKM stages changes over time during the follow-up period among those with 2^nd^ visit.

Abbreviations: CKM: cardiovascular–kidney–metabolic syndrome; N: number of participants; HR: hazard ratio; CI: confidence interval; Ref: reference group; Ref: reference group.
