# Supplementary material for: Cardiovascular–kidney–metabolic syndrome and all-cause and cardiovascular mortality: A retrospective cohort study
Source: PLoS Med. 2025 Jun 26;22(6):e1004629. doi: 10.1371/journal.pmed.1004629 (PMC12200875; doi:10.1371/journal.pmed.1004629)
Supplement: S1 Fig — (DOCX) [file pmed.1004629.s013.docx]

**
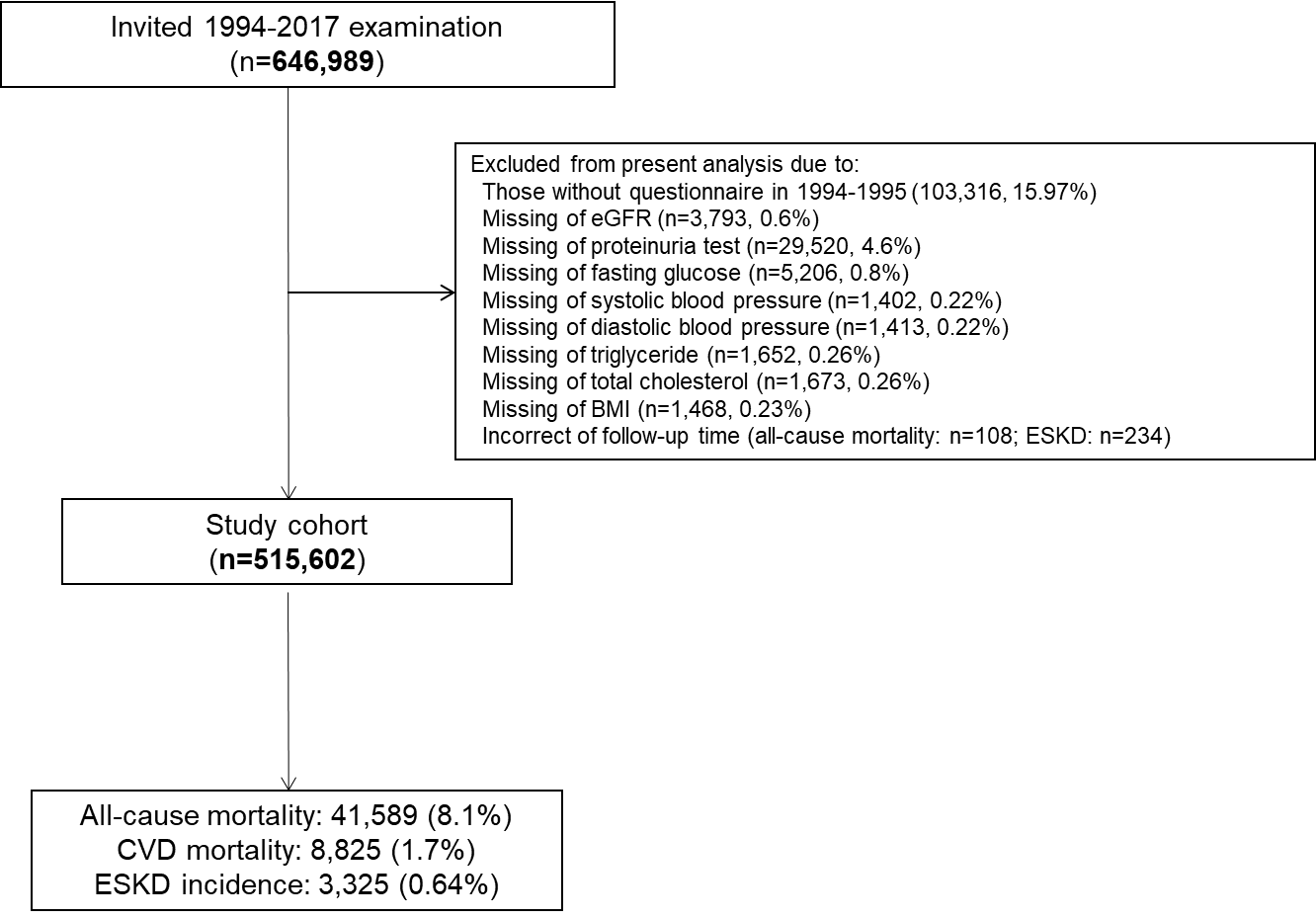
**

# Figure S1. Participants selection process

Abbreviations: eGFR: estimated glomerular filtration rate; N: number of participants; BMI: body mass index; ESKD: end-stage kidney disease; CVD: cardiovascular disease.
