# Supplementary material for: Cardiovascular–kidney–metabolic syndrome and all-cause and cardiovascular mortality: A retrospective cohort study
Source: PLoS Med. 2025 Jun 26;22(6):e1004629. doi: 10.1371/journal.pmed.1004629 (PMC12200875; doi:10.1371/journal.pmed.1004629)
Supplement: S2 Fig — (DOCX) [file pmed.1004629.s014.docx]

**
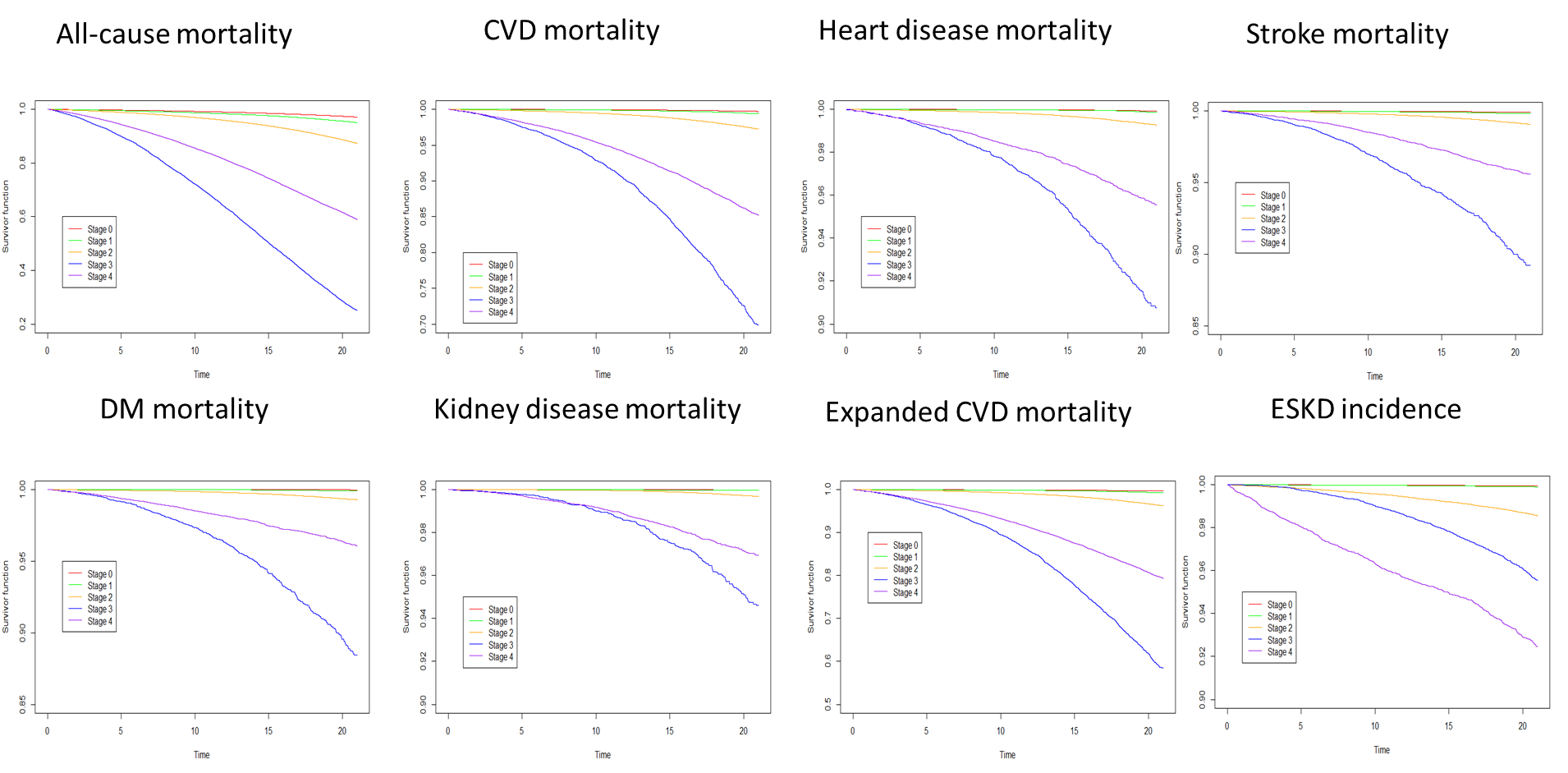
**

# Figure S2. Kaplan–Meier survival curves stratified by cardiovascular–kidney–metabolic syndrome stage

Abbreviations: CVD: cardiovascular disease; DM: diabetes mellitus; ESKD: end-stage kidney disease.
