# Supplementary material for: Cardiovascular–kidney–metabolic syndrome and all-cause and cardiovascular mortality: A retrospective cohort study
Source: PLoS Med. 2025 Jun 26;22(6):e1004629. doi: 10.1371/journal.pmed.1004629 (PMC12200875; doi:10.1371/journal.pmed.1004629)
Supplement: S4 Fig — (DOCX) [file pmed.1004629.s016.docx]

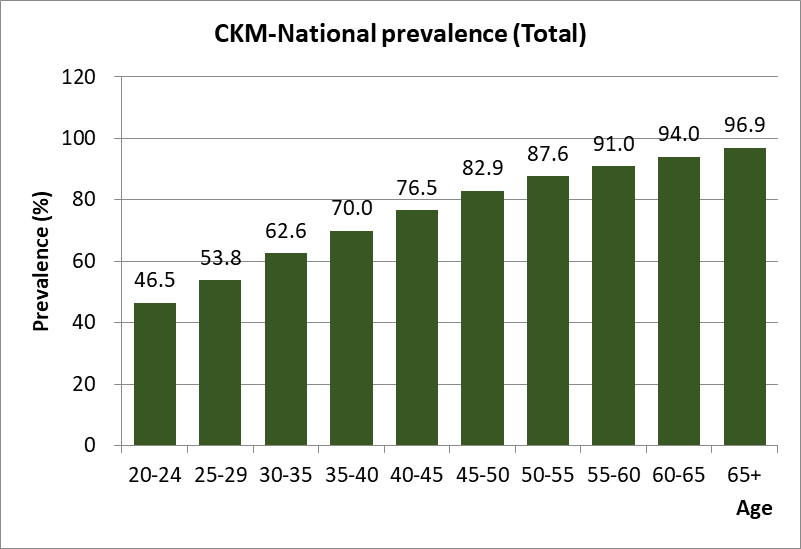

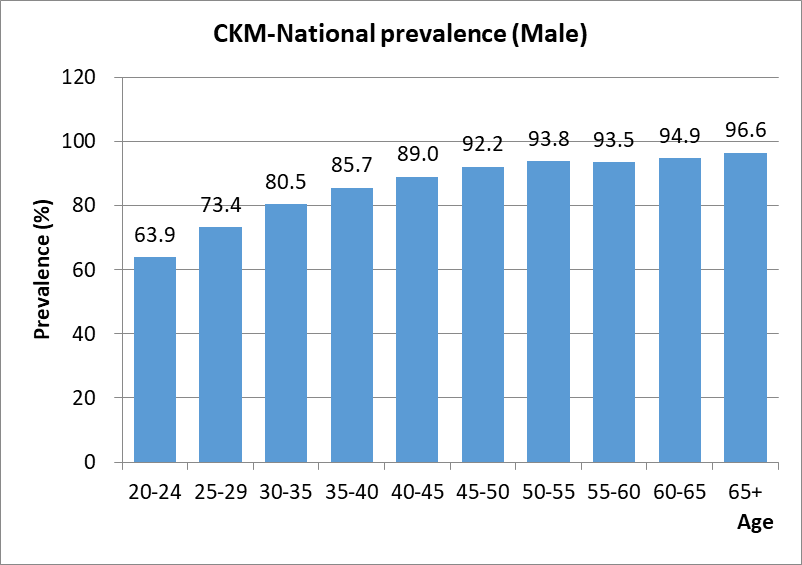

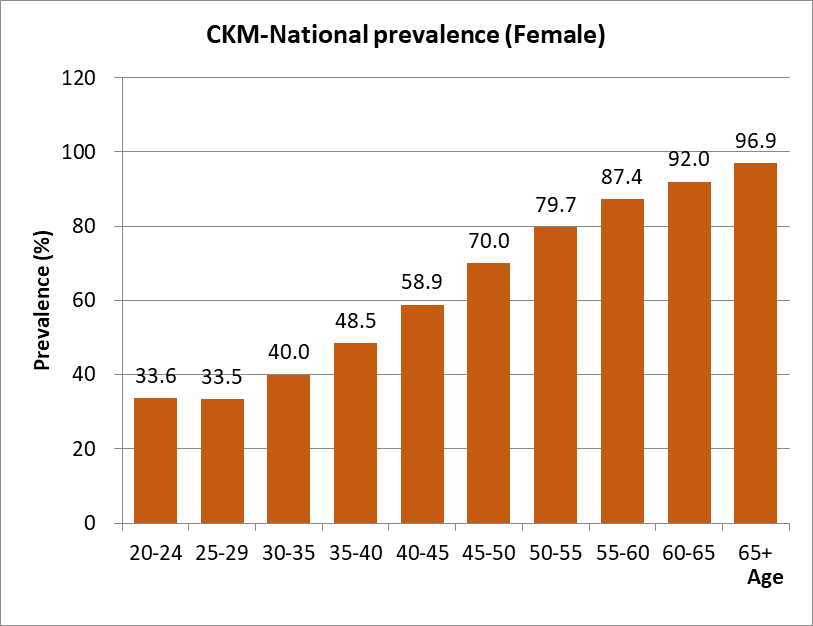


# Figure S4. Age and education-adjusted CKM prevalence

Abbreviations: CKM: cardiovascular–kidney–metabolic syndrome;
