# Supplementary material for: Cardiovascular–kidney–metabolic syndrome and all-cause and cardiovascular mortality: A retrospective cohort study
Source: PLoS Med. 2025 Jun 26;22(6):e1004629. doi: 10.1371/journal.pmed.1004629 (PMC12200875; doi:10.1371/journal.pmed.1004629)
Supplement: S6 Fig — (DOCX) [file pmed.1004629.s018.docx]

**
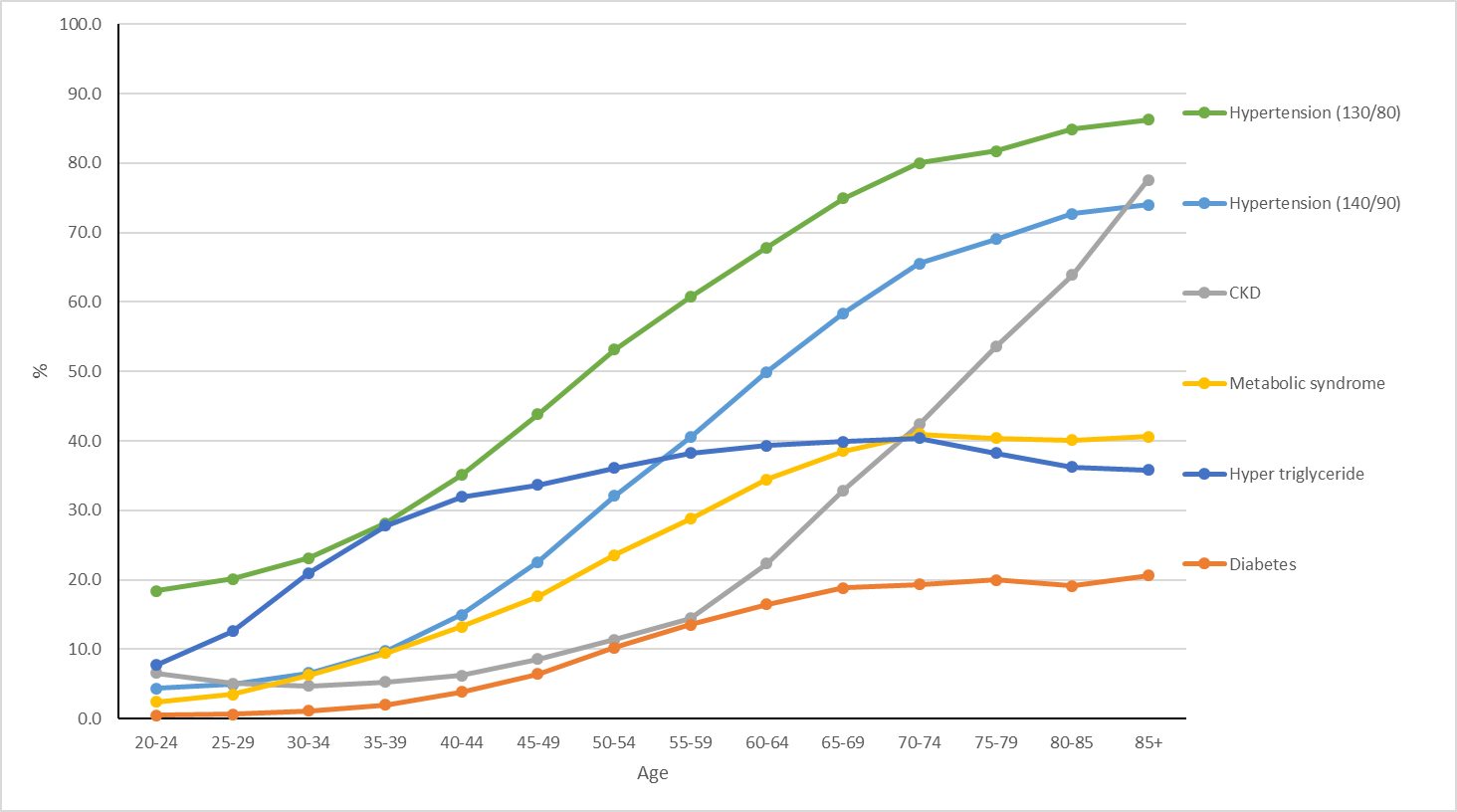
**

# Figure S6. Age-specific prevalence of hypertension, chronic kidney disease, diabetes, metabolic syndrome, and hypertriglycerides in study cohort

Abbreviations: CKD: Chronic kidney disease.
