# Supplementary material for: Cardiovascular–kidney–metabolic syndrome and all-cause and cardiovascular mortality: A retrospective cohort study
Source: PLoS Med. 2025 Jun 26;22(6):e1004629. doi: 10.1371/journal.pmed.1004629 (PMC12200875; doi:10.1371/journal.pmed.1004629)
Supplement: S2 File — (DOCX) [file pmed.1004629.s020.docx]

# Supplement material 2: SAS code for life-table methods

options ls=80 ps=60;

data _NULL_;

/* C:\TSAI\SASLIFE.PRG */

/* ABRIDGED LIFE TABLE PROGRAM */

/* ------------------- */

/* */

/* INPUT STARTS WITH 16 CARDS AS FOLLOWS : */

/* 1. TITLE CARD, 1 CARD 80 Columns */

/* 2. TOTALS CARD, 1 CARD IN FORMAT(I4,5X,I9,F9.0,I9,F9.0) */

/* CONTAINING THE YEAR (IDATE) IN COLUMNS 1-4 */

/* TOTAL POPULATION (ITPP) IN COLUMNS 10-18 */

/* TOTAL DEATHS (TDD) IN COLUMNS 19-27 */

/* THE NO. OF AGE GROUPS (IC) IN COLUMNS 28-36 */

/* Note: IC is the number of Age Groups including */

/* the Unknown Group - the Unknown Group */

/* should always be the last Age Group */

/* and must be entered as Zeros if no Unknowns */

/* THE NO. OF YEARS TO WHICH DEATH DATA APPLY (YEARS) */

/* IN COLUMNS 37-45. IF THEN NUMBER OF YEARS IS NOT PUNCHED*/

/* THE DEFAULT VALUE IS ONE YEAR. */

/* 3. TOTAL POPULATION BY AGE (IPP), 2 CARDS IN FORMAT (9I8) */

/* Note: Remember 0 for last group if no Unknown */

/* 4. TOTAL DEATHS BY AGE (DD), 2 CARDS IN FORMAT (9F8.0) */

/* Note: Remember 0 for last group if no Unknown */

/* ================================================================= */

/* Optional Cards - See Below */

/* ----------------------------------------------------------------- */

/* 5. THE FRACTION OF LAST YEAR OF LIFE (AX) FOR AGES 0-4 */

/* FOR TOTAL POPULATION (A(0)=.10,A(1)=.43,A(2)=.45,A(3)=.47*/

/* A(4)=.49. A(0)=.14 FOR BLACK, 1 CARDS IN FORMAT (5F5.2) */

/* 2 Cards in Format (9F8.0 - Decimal Point is Boss) */

/* Read in 18 of them even if you need less */

/* 6. PROPORTION DYING (QXC) FROM PREVIOUS PUBLISHED COMPLETE */

/* LIFE TABLE (AGE 0 UP TO 85), */

/* AGE GROUPS ARE : 1,1-4,5-9,...,80-84, 85+, AND UNKNOWN */

/* (20 CATEGORIES). NUMBER OF CATEGORIES CAN BE VARIED. */

/* 2 Cards in Format (9F8.0 - Decimal Point is Boss) */

/* Read in 18 of them even if you need less */

/* ================================================================= */

/* SETS OF THE ABOVE INPUT MAY BE REPEATED INDEFINITELY. */

/* ================================================================= */

/* LIFE TABLE VARIABLES : */

/* A(I)=FRACTION OF LAST AGE INTERVAL OF LIFE */

/* QX(I)=PROPORTION DYING IN INTERVAL */

/* DX(I)=NUMBER DYING IN INTERVAL */

/* VMX(I)=AGE-SPECIFIC DEATH RATES */

/* VLX(I)=NUMBER LIVING AT AGE X(I) */

/* VLLX(I)=NUMBER OF YEARS LIVED IN INTERVAL */

/* TX(I)=TOTAL NUMBER OF YEARS LIVED BEYOND AGE X(I) */

/* EX(I)=OBSERVED EXPECTATION OF LIVE AT AGE X(I) */

/* STER(I)= STANDARD DEVIATION OF PROPORTION DYING IN INTERVAL */

/* STEX(I)= STANDARD DEVIATION OF OBSERVED EXPECTATION OF LIFE */

/* AT AGE X(I) */

/* ------------------------------------------------------------------*/

/* From Tsai's Original Program */

/* DIMENSION NAME(19), PP(20), DD(20), IAGE(19), IPP(20), */

/* 1 VMX(20), A(20), QX(20), IDX(20), VLX(20), VLLX(20), */

/* 2 TX(20), IDD(20), EX(20), LX(20), DX(20), LLX(20), */

/* 3 ITX(20), VARQ(19), STER(19), SSEX(19), PB(19,19), */

/* 4 SEXI(19,19), STEX(19); */

/* ================================================================= */

array pp(20) pp01-pp20;

array dd(20) dd01-dd20;

array iage(19) iage01-iage19;

array ipp(20) ipp01-ipp20;

array vmx(20) vmx01-vmx20;

array a(19) a01-a19;

array qx(20) qx01-qx20;

array vlx(20) vlx01-vlx20;

array vllx(20) vllx01-vllx20;

array tx(20) tx01-tx20;

array ex(20) ex01-ex20;

array dx(20) dx01-dx20;

array varq(19) varq01-varq19;

array ster(19) ster01-ster19;

array ssex(19) ssex01-ssex19;

array stex(19) stex01-stex19;

array pb(19,19) pb001-pb361;

array sexi(19,19) sexi001-sexi361;

/* INPUT DATA */

input name $char80.;

input idate 1-4 itpp 10-18 tdd 19-27 ic 28-36 years 37-45;

input @1 (ipp01-ipp10) (8.0) / @1 (ipp11-ipp15) (8.0);

input @1 (dd01-dd10) (8.0) / @1 (dd11-dd15) (8.0);

Interval=5; /* Set Interval Size */

FILE PRINT;

IC1 = IC - 1;

IC2 = IC - 2;

IF (YEARS <= 0 OR YEARS = 1) then GO TO num28;

RECIP = 1.0/YEARS;

DO I = 1 to IC;

DD(I) = DD(I)*RECIP;

end;

/* DEATHS HAVE BEEN ADJUSTED TO A ONE YEAR INTERVAL */

/* CHECK TO ASCERTAIN IF EACH TOTAL EQUALS THE SUM OF THE AGES */

num28: LPP = 0;

ADD = 0.;

DO I = 1 to IC;

LPP = LPP + IPP(I);

ADD = ADD + DD(I);

end;

ID1 = LPP - ITPP;

ID2 = ADD - TDD;

IF ((ABS(ID1) + ABS(ID2)) < 1) then GO TO num30;

put /// @1 'Discrepancy in Population is ' id1/

@1 'Discrepancy in Deaths is ' id2;

GO TO num58;

/* NEXT, PRINT THE DATA */

/* FIRST, SET UP THE STUB OF THE TABLE */

num30: IAGE(1) =20;

DO I =2 to IC1; /* Remember last group is Unknown group */

IAGE(I) = IAGE(I-1) + Interval;

end;

*IAGE(1)=0;

*IAGE(2)=1;

/* PRINT THE TABLES */

put _page_;

put /

@1 ' AGE' @13 ' POPULATION' @31 ' DEATHS'

/ @1 ' ===' @13 ' ==========' @31 ' ======';

do i=1 to ic1;

put @1 iage(i) 7.0 @13 ipp(i) 12.0 @31 dd(i) 12.0;

end;

put @1 'Unknown' @13 ipp(ic) 12.0 @31 dd(ic) 12.0;

put @1 @13 ' ----------' @31 ' -------'/

@1 ' Total' @13 itpp 12.0 @31 tdd 12.0;

/* DISTRIBUTE THE UNKNOWN CATEGORY OF POPULATION */

DO I = 1 to IC1;

IPP(I) =(IPP(I)*ITPP/(ITPP - IPP(IC)))+0.5;

DD(I) = (DD(I)*TDD/(TDD - DD(IC)))+0.5;

VMX(I) = DD(I)/IPP(I);

end;

* vmx(13)=vmx(12)*1.5;

vmx(14)=vmx(13)*7;

/* COMPUTE AND PRINT THE ABRIDGED LIFE TABLE */

Put / @1 name $char80.;

/*==================================================================*/

/* If input of A's and/or QX's - do it here - remove comment */

/*------------------------------------------------------------------*/

/* */

* input @1 (a01-a09) (8.0) / @1 (a10-a18) (8.0);

* input @1 (qx01-qx09) (8.0) / @1 (qx10-qx18) (8.0);

/*==================================================================*/

/* If you input A's and/or QX's - then comment out the following */

/* Otherwise let them be calculated as below: */

/*------------------------------------------------------------------*/

DO I = 1 to IC1;

A(I) =0.50; /* Fraction of last age interval = 0.5 */

end;

* A(1)=0.11;

*A(2)=0.38;

*A(3)=0.45;

DO I = 1 to IC2;

QX(I) = Interval*VMX(I)/(1. + (1. - A(I))*Interval*VMX(I));

end;

* QX(1) = 1*VMX(1)/(1. + (1. - A(1))*1*VMX(1));

* QX(2) = 4*VMX(2)/(1. + (1. - A(2))*4*VMX(2));

QX(IC1) = 1.;

/*==================================================================*/

/* Want to set some QX's - do it below - remove comments */

/*------------------------------------------------------------------*/

* QX(12) = 0.2365;

* QX(13) = 0.3229;

* QX(14) = 0.1662;

/*------------------------------------------------------------------*/

VLX(1) = 100000.; /* Set Radix */

DO I = 1 to IC2;

DX(I) = VLX(I)*QX(I);

VLX(I+1) = VLX(I) - DX(I);

VLLX(I) = Interval*(VLX(I) - DX(I)) + Interval*A(I)*DX(I);

end;

*VLLX(1) = 1*(VLX(1) - DX(1)) + 1*A(1)*DX(1);

* VLLX(2) = 4*(VLX(2) - DX(2)) + 4*A(2)*DX(2);

VLLX(IC1) = VLX(IC1)/VMX(IC1);

DX(IC1) = VLX(IC1);

TX(IC1) = VLLX(IC1);

EX(IC1) = TX(IC1)/VLX(IC1);

DO I = 1 to IC2; /* Change */

L = IC1 - I;

M = IC - I;

TX(L) = TX(M) + VLLX(L);

EX(L) = TX(L)/VLX(L);

end;

DO I = 1 to IC2;

VARQ(I) = (1/DD(I))*QX(I)*(1-QX(I));

STER(I) = SQRT(VARQ(I));

end;

varq(ic1)=0;

ster(ic1)=0;

DO K = 1 to IC2;

SSEX(K) = 0.;

DO I = K to IC2;

PB(K,I) = VLX(I)/VLX(K);

SEXI(K,I)=(PB(K,I)*PB(K,I)*

((EX(I+1)+(1 -A(I))*5)**2)*VARQ(I));

SSEX(K) = SSEX(K) + SEXI(K,I);

end;

STEX(K) = SQRT(SSEX(K));

end;

stex(ic1)=0;

put /@1 'AGE' @13 'PP' @22 'DD' @32 'Q(X)' @42 'L(X)'

@52 'D(X)' @61 'LL(X)' @70 'AGE'/

@1 '===' @13 '==' @22 '==' @32 '====' @42 '===='

@52 '====' @61 '=====' @70 '===';

do i=1 to ic1;

put @1 iage(i) 3.0 ' ' ipp(i) 9.0 dd(i) 9.0

qx(i) 12.6 vlx(i) 10.0 dx(i) 10.0 vllx(i) 10.0

' ' iage(i) 4.0;

end;

put ////////////@1 'AGE' @11 'M(X)' @20 'A(X)' @31 'TT(X)' @42 'E(X)'

@49 'STDQ(X)' @59 'STDE(X)' @70 'AGE'/

@1 '===' @11 '====' @20 '====' @31 '=====' @42 '===='

@49 '=======' @59 '=======' @70 '===';

do i=1 to ic1;

put @1 iage(i) 3.0 ' ' vmx(i) 9.6 a(i) 9.2 tx(i) 12.0

ex(i) 10.2 ster(i) 10.6 stex(i) 10.2 ' ' iage(i) 4.0;

end;

num58: return;

cards;

==> CKM stage 0 male LIFE EXPECTANCY 2025

2025 779172 1490 15 1

8125 50340 108995 141902 143766 117461 79603 49225 30191 19933

13797 9026 4882 1926 0

8 25 38 89 95 115 136 89 117 128

159 187 163 141 0

;

run;
