# Supplementary material for: Cardiovascular–kidney–metabolic syndrome and all-cause and cardiovascular mortality: A retrospective cohort study
Source: PLoS Med. 2025 Jun 26;22(6):e1004629. doi: 10.1371/journal.pmed.1004629 (PMC12200875; doi:10.1371/journal.pmed.1004629)
Supplement: Attachment — Submitted filename: Response_Reviewer_Comments_20250221.docx [file pmed.1004629.s023.docx]

**Comments from the academic editor:**

My impression is that there are mixed feelings about the CKM syndrome and a sense of skepticism about the usefulness of AHA-defined syndromes in the clinical community. It will be important for the authors to make clear how the conceptual model of this type of research helps us to improve health care and health. The CKM stages are defined by progressively more severe organ dysfunction - was there any doubt about the findings? The authors should discuss the novelty of their findings more clearly, e.g. one could argue that it is well known that risks accumulate so that they increase as a person has more diseases. The aim of the paper should also be communicated more clearly, whether it is to predict mortality or disease events, to create clusters of patient types, or to investigate causal inference. In short, the manuscript would benefit from a stronger presentation with clearer rationale and goals.

***Response:***

Thank you for the comments.

In response to the feedback provided by the editor and reviewers, we have thoroughly revised our manuscript. We have incorporated the relevant analytical results as requested. Additionally, to enhance the clarity of our rationale and research goals, we have revised the introduction as follows.

**Introduction, 2^nd^ paragraph, Line 77:**

“In November 2023, the American Heart Association (AHA) published new guidelines that emphasize the importance of cardiovascular–kidney–metabolic (CKM) health for the first time [8, 9]. Patients with CVD often have comorbid hypertension, kidney disease, and metabolic disorders, which share common underlying mechanisms and have a critical impact on CVD incidence and mortality [10-13]. This CKM syndrome is defined and staged based on the presence of clinical characteristics (e.g., obesity) and comorbidities (e.g., hypertension, diabetes, and chronic kidney disease (CKD)). Because CKM syndrome is a newly defined concept, it is essential to understand its impact on mortality risk and CVD outcomes, as well as the distribution of different CKM stages in the general population. Additionally, CKM introduces an interdisciplinary approach to health, shifting the focus from individual disease factors to the interactions among multiple diseases. CKM syndrome emphasize the importance of multidisciplinary approaches to prevention, risk stratification, and treatment for these diseases. Unlike previous models for CVD risk prediction, CKM syndrome incorporates kidney disease-related risk factors. Recently, international scientific societies have advocated for the inclusion of kidney disease in the World Health Organization’s (WHO) framework on major non-communicable disease (NCD) determinants of premature mortality [14]. However, while incorporating CKD into this framework acknowledges its significance, concerns remain regarding its predictive utility for all-cause and CVD outcomes, particularly given the limited public awareness of CKD[15]. Recent therapeutic advances further support the rationale for an integrated CKM framework, as several novel medications demonstrate beneficial effects across multiple disease domains [16]. Sodium-glucose cotransporter-2 (SGLT2) inhibitors, for example, have shown remarkable efficacy in reducing cardiovascular events, slowing CKD progression, and improving glycemic control [17]. Similarly, glucagon-like peptide-1 (GLP-1) receptor agonists [18] and nonsteroidal mineralocorticoid receptor antagonists (MRAs) exhibit protective effects across multiple organ systems [19]. These findings suggest that optimal treatment strategies should consider the entire CKM spectrum rather than focusing on isolated conditions.

Therefore, this study investigates the combinations of different CKM components and their association with mortality risk, including hypertension, chronic kidney disease, diabetes mellitus, metabolic syndrome, and hyperlipidemia. Previously, we used a large cohort to examine the prevalence and mortality burden of CKD [20]. We also accessed the public awareness of each of the CKM components. In the present study, we investigated the risks of all-cause mortality, CVD mortality, and ESKD associated with various CKM components. Additionally, we examined the effects of individual and multiple CKM components on mortality and ESKD risk.”

**Comments from the editorial team:**

We request that you include a more detailed description of the study cohort in the main text of the manuscript, as we feel that the references to the previous reports are not sufficient.

***Response:***

We agreed with editors’ suggestion. We have added a detailed description of our study cohort in the Methods section:

**Page 7, Line 123**

“The study cohort was derived from a private health screening program in Taiwan. Standardized screening procedures were implemented using identical instruments, ensuring uniformity in data collection [20, 21]. All results were centrally managed and securely stored. Participants were non-institutionalized, generally healthy individuals from diverse backgrounds. They were encouraged to undergo annual health screenings, during which identical questionnaires were administered at each visit. However, for this study, only baseline data from the initial visit were used for analysis. This study was approved by the Institutional Review Board of Taipei Medical University, and details of the cohort have been previously reported [21, 22]. All personally identifiable information was removed, and data remained anonymized throughout the study process.”

**Comments from the reviewers:**

Reviewer #1: Following the recent American Heart Association (AHA) publication defining Cardiovascular-Kidney-Metabolic (CKM) Syndrome, this research aimed to use the AHA CKM syndrome definition to understand both prevalence of CKM and associations between CKM severity and serious outcomes in an Asian cohort. The authors primarily used Cox regression adjusted for baseline characteristics to understand the associations between various operationalisations of CKM (e.g., CKM severity stages 1-4, types of diseases constituting CKM, number of CKM diseases present) and various mortality endpoints plus end-stage kidney disease. They found most of the population examined had some level of CKM and, perhaps unsurprisingly, increasing severity of CKM led to worse outcomes.

This is an interesting paper that is broadly well presented and important in the context of CKM syndrome emerging as a new biomedical and public health concept. I have concerns about the lack of methodological detail provided - particularly important when analysing observational cohorts - and the bulk of my feedback below seeks to elicit this detail, but I am confident this is fully addressable by the authors. I am more troubled by a substantial limitation that I feel has not yet been sufficiently described and addressed by the authors. The issue is all analyses appear to be based on baseline data. That is, if a participant was recruited when they were healthy and had no CKM-defining disease states, they were assumed to be disease-free until they had an endpoint recorded. Similarly for patients having more (or even less) severe CKM over time. There is an obvious risk of misclassification bias as patients are followed-up over the long term to death. I have provided more detail on this below, but this must be called out early and a cogent argument presented to convince readers the analyses presented are valid.

Detailed feedback on the manuscript is provided below. My primary focus has been methodological, but I have made a few broader points as well. All items are major unless indicated otherwise. While I avoid "nitpicking" language used, there were a couple of cases (1 & 2 below) where I was troubled by the words chosen in prominent positions, felt they may be misleading, and thought it worth noting.

***Response:***

Thank you for the comments. We have incorporated additional sensitivity analyses to further support the robustness of our findings. To enhance clarity and strengthen the presentation of our study, we have also revised the Introduction and Methods sections to better articulate our rationale and research objectives.

1. [MINOR] The title wording is confusing to me. Suggest rewording - especially reconsider the use of "Unveiling the creation…" as this doesn't really make sense in the context of the paper.

***Response:***

We have revised our title accordingly:

“The Cardiovascular-Kidney- Metabolic Syndrome: the Contribution to All-Cause and Cardiovascular Mortality”.

2. [MINOR] Similarly, the use of the word "mitigate" in the first sentence of the abstract is confusing.

***Response:***

We have revised this sentence.

**Page 3, Abstract, Line 3**

“The American Heart Association recently issued guidelines introducing the concept of cardiovascular–kidney–metabolic (CKM) syndrome to emphasize the importance of multidisciplinary approaches to prevention, risk stratification, and treatment for these diseases. This study assessed the prevalence of CKM syndrome stages and the mortality risk associated with its components in a large Asian cohort.”

3. [MINOR] Please ensure all abbreviations are defined when first used. "CKM" is used in the second para of the Introduction but is not defined until the third para.

***Response:***

We have checked all abbreviations when first used.

4. The Introduction would benefit from being more focussed on defining CKM syndrome and establishing the motivation for this research clearly and succinctly. The first paragraph is fine, but the second seems to be extraneous to the purpose of this paper i.e., quantifying the prevalence and mortality associations of CKM syndrome in a large observational cohort does not require the historical development of CKM. I would also reword the third paragraph to be clearer, more focussed, and as succinct as possible e.g., "In November 2023, the American Heart Association (AHA) published new guidelines that defined cardiovascular-kidney-metabolic (CKM) syndrome for the first time. CKM syndrome is defined and staged based on the presence of clinical characteristics (e.g. obesity) and comorbidities (e.g., CVD, CKD). Because CKM syndrome is a newly defined concept…" Note I am not suggesting the authors must use this example wording, rather I hope it provides a helpful illustration of one

way to improve clarity.

***Response:***

We agreed with reviewer’s suggestion. We have revised the introduction.

**Introduction, 2^nd^ paragraph, Line 77**

“In November 2023, the American Heart Association (AHA) published new guidelines that emphasize the importance of cardiovascular–kidney–metabolic (CKM) health for the first time [8, 9]. Patients with CVD often have comorbid hypertension, kidney disease, and metabolic disorders, which share common underlying mechanisms and have a critical impact on CVD incidence and mortality [10-13]. This CKM syndrome is defined and staged based on the presence of clinical characteristics (e.g., obesity) and comorbidities (e.g., hypertension, diabetes, and chronic kidney disease (CKD)). Because CKM syndrome is a newly defined concept, it is essential to understand its impact on mortality risk and CVD outcomes, as well as the distribution of different CKM stages in the general population. Additionally, CKM introduces an interdisciplinary approach to health, shifting the focus from individual disease factors to the interactions among multiple diseases. CKM syndrome emphasize the importance of multidisciplinary approaches to prevention, risk stratification, and treatment for these diseases. Unlike previous models for CVD risk prediction, CKM syndrome incorporates kidney disease-related risk factors. Recently, international scientific societies have advocated for the inclusion of kidney disease in the World Health Organization’s (WHO) framework on major non-communicable disease (NCD) determinants of premature mortality [14]. However, while incorporating CKD into this framework acknowledges its significance, concerns remain regarding its predictive utility for all-cause and CVD outcomes, particularly given the limited public awareness of CKD[15]. Recent therapeutic advances further support the rationale for an integrated CKM framework, as several novel medications demonstrate beneficial effects across multiple disease domains [16]. Sodium-glucose cotransporter-2 (SGLT2) inhibitors, for example, have shown remarkable efficacy in reducing cardiovascular events, slowing CKD progression, and improving glycemic control [17]. Similarly, glucagon-like peptide-1 (GLP-1) receptor agonists [18] and nonsteroidal mineralocorticoid receptor antagonists (MRAs) exhibit protective effects across multiple organ systems [19]. These findings suggest that optimal treatment strategies should consider the entire CKM spectrum rather than focusing on isolated conditions.”

5. I appreciate cohort details have been reported previously, but I think a little more detail is needed in this paper. Specifically, how were participants recruited, when were the tests and questionnaires completed (once at baseline? repeatedly throughout follow-up?), characterising of follow-up (at least mean follow-up), and was there any in-person follow-up or was follow-up only done by linkage to deaths?

***Response:***

Participants were non-institutionalized, generally healthy individuals from diverse backgrounds. They were encouraged to undergo annual health screenings, during which identical questionnaires were administered at each visit. However, for this study, only baseline data from the initial visit were used for analysis. The follow-up was done by linkage to deaths.

We have added a detailed description of our study cohort in the Methods section.

**Page 7, 2^nd^ paragraph, Line 123**

“The study cohort was derived from a private health screening program in Taiwan. Standardized screening procedures were implemented using identical instruments, ensuring uniformity in data collection [20, 21]. All results were centrally managed and securely stored. Participants were non-institutionalized, generally healthy individuals from diverse backgrounds. They were encouraged to undergo annual health screenings, during which identical questionnaires were administered at each visit. However, for this study, only baseline data from the initial visit were used for analysis. This study was approved by the Institutional Review Board of Taipei Medical University, and details of the cohort have been previously reported [21, 22]. All personally identifiable information was removed, and data remained anonymized throughout the study process.”

Taiwan maintains a death registry on the basis of citizen death certificates. We linked the unique identification number to this database to obtain mortality outcomes.

The follow-up information was provided as follows.

**Page 10, 2^nd^ paragraph, Line 240**

“A total of 41,589 all-cause deaths, 8,825 CVD deaths, and 3,334 incidences of ESKD were identified over a median follow-up period of 16.5 years (interquartile range, IQR: 11.5–21.2).”

6. Relatedly, how do the analyses account for changes in an individual's health status over time e.g., a patient who changes CKM stage due to development of a disease like hypertension, CKD, etc.?

***Response:***

We acknowledge that CKM stages may change over time. In this study, we used CKM stage at baseline to predict mortality risk, rather than analyzing individual-level changes in CKM stage over time. However, we conducted an analysis among participants who had undergone two health examinations, using their second CKM stage assessment to evaluate differences in mortality risk.

Among participants with a second examination, the prevalence of CKM stages was similar to that at baseline (prevalence in the second examination: CKM stage 1: 21.1%; stage 2: 45.3%; stage 3: 1.6%; stage 4: 4.1% vs. baseline CKM prevalence: CKM stage 1: 19.5%; stage 2: 46.3%; stage 3: 1.9%; stage 4: 3.8%). Regarding the association between CKM and mortality risk, the hazard ratios among individuals with a second examination were as follows: CKM stage 1: 0.87; stage 2: 1.23; stage 3: 1.75; stage 4: 2.10, compared to the baseline CKM hazard ratios: CKM stage 1: 0.96; stage 2: 1.36; stage 3: 2.13; stage 4: 2.37.

Additionally, we have incorporated a time-dependent analysis for participants with a second examination as a sensitivity analysis. This approach accounts for changes in CKM stages over time and the duration of follow-up, allowing us to further evaluate the association between CKM stages and mortality outcomes.

Furthermore, in the study limitations, we have suggested that future research should focus on the impact of CKM stage changes over time to provide a more comprehensive understanding of its implications.

Limitation:

**Page 16, Line 435**

“Third, our analysis was based only on baseline measurements, which do not account for changes in individual circumstances over time. Future research should focus on the impact of CKM stage changes over time to provide a more comprehensive understanding of its implications. However, we conducted an analysis among participants who had undergone two health examinations, using their second CKM stage assessment to evaluate differences in mortality risk. We also incorporated a time-dependent analysis, and this yielded similar results (Table S12).”

7. Please define CKM Stage 0 because the authors use this later in the paper. Consider including in the paper/supplement something similar to "Table 3. Definitions of CKM Health Stages" from the paper's reference 20. Being able to see the CKM syndrome stages easily in a table is very helpful.

***Response:***

We have added a new table for CKM definitions.

Table S1. Definition of CKM syndrome stages in this study

| **CKM Syndrome Stages** | **Definition** |  |
| --- | --- | --- |
| Stage 0: No CKM risk factors | Individuals with normal BMI and waist circumference, normoglycemia, normotension, a normal lipid profile, and no evidence of CKD or subclinical or clinical CVD |  |
| Stage 1: Excess or dysfunctional adiposity | Individuals with overweight/obesity, abdominal obesity, or dysfunctional adipose tissue, without the presence of other metabolic risk factors or CKD BMI^a^ ≥ 23 kg/m^2^,  waist circumference^a^ ≥ 80 cm for women and ≥ 90 cm for men, or  fasting blood glucose level = 100–124 mg/dL.^b^ |  |
| Stage 2: Metabolic risk factors and CKD | Presence of hypertension,^c^  diabetes,^d^  metabolic syndrome,^e^  high triglyceride levels (≥ 135 mg/dL),^f^ or  any stage of CKD.^g^ |  |
| Stage 3: Subclinical CVD in CKM | With a Predicting Risk of cardiovascular disease EVENTs (PREVENT) score indicating a 10-year risk of CVD exceeding 20% or  has a kidney function with an eGFR of ≤30 mL/min/1.73 m². |  |
| Stage 4: Clinical CVD in CKM | Self-reports medication use or seeks medical attention for coronary heart disease, heart failure, or stroke or  has CKD with an eGFR of ≤30 mL/min/1.73 m^2^. |  |
| ^a^ Asian criteria  ^b^ HbA1c are not available in this cohort  ^c^ Hypertension was defined as a history of hypertension, the use of antihypertensive medication, or a systolic blood pressure of ≥130 mmHg  or a diastolic blood pressure of ≥80 mmHg.  ^d^ Diabetes was defined as a history of diabetes, the use of diabetes medication, or a fasting blood glucose level of ≥126 mg/dL.  ^e^ Metabolic syndrome was defined as the presence of at least three of the following criteria: waist circumference≥80 cm for women and ≥90 cm for men;  systolic blood pressure ≥130 mmHg or diastolic blood pressure ≥80 mmHg and/or use of antihypertensive medications;  fasting blood glucose ≥100 mg/dL;  HDL cholesterol <40 mg/dL for men and <50 mg/dL for women;  triglycerides ≥150 mg/dL.  ^f^ High triglyceride levels was defined as a history of hyperlipidemia, the use of lipid-lowering medication, or a triglyceride level of ≥135 mg/dL.  ^g^ CKD was determined on the basis of eGFR by using the CKD Epidemiology Collaboration (CKD-EPI) formula,  and proteinuria levels were detected using a dipstick test, categorized as negative, trace, 1+, 2+, or 3+.  ^h^ Calculated by the equation of PREVENT exceeding 20% risk | | |

8. Relatedly, it's extremely important to be clear on whether the authors are using the AHA's definitions or if they are adapting the AHA's definitions for this research. For example, in reference 20 the AHA includes "or HbA1c between 5.7% and 6.4%" in their definition of Stage 1 but it is omitted from the Stage 1 definition in this paper. I am not sure if this is a typo or if the authors are adapting the AHA's definition for clinical or logistical reasons.

***Response:***

In this study, diabetes was defined as a history of diabetes, the use of diabetes medication, or a fasting blood glucose level of ≥126 mg/dL.HbA1c are not available in this cohort. We have defined this in the definition table S1.

9. How did the authors account for the use of antihypertensives for indications outside of hypertension e.g., migraine prophylaxis?

***Response:***

Participants who self-reported the use of antihypertensive medication or a history of hypertension were classified under the hypertension component. Additionally, we conducted a sensitivity analysis considering the use of analgesics. After incorporating the variable “analgesic use,” the main results, including all-cause mortality and cardiovascular mortality, remained unchanged.

**Page 11, Line 270**

“Additionally, we conducted a sensitivity analysis considering the use of analgesics (2.1%) and cancer history (1.1%). After incorporating the variables, the main results, including all-cause mortality and cardiovascular mortality, remained unchanged (Table S4).”

10. Why was reference 26 included? It appears unrelated to the sentence it was cited in.

***Response:***

We have deleted this reference.

11. [MINOR] While hazard ratios are the generally accepted statistic for time to event analyses, they have some limitations (refer DOIs: 10.1093/ehjacc/zuae017 and 10.1056/EVIDe2300142). Consider supplementing the key HR outcomes with another statistic like difference in median survival or RMST.

***Response:***

Thank you to the reviewer for the valuable suggestions and for providing the relevant literature. When the proportional hazards assumption is not met, using RMST is a more appropriate approach. We additionally performed RMST regression for each CKM stage. The results showed that as CKM stage and the number of CKM components increased, RMST values progressively decreased. Furthermore, we found consistent results for both all-cause mortality and cardiovascular disease mortality. This analysis further strengthens the validity of our findings.

Table S2. Restricted mean survival time regression model for all-cause and CVD mortality by cardiovascular–kidney–metabolic syndrome stage

| CKM | RMST | RMST difference | (95% CI) | |
| --- | --- | --- | --- | --- |
| Stage 0 | 23.395 |  |  |  |
| Stage 1 | 23.248 | -0.147 | (-0.19 | ,-0.11) |
| Stage 2 | 23.140 | -0.255 | (-0.29 | ,-0.22) |
| Stage 3 | 21.161 | -2.234 | (-2.44 | ,-2.03) |
| Stage 4 | 19.043 | -4.352 | (-4.72 | ,-3.98) |
| All CKM |  |  |  |  |
|  |  |  |  |  |
| Zero components | 23.159 |  |  |  |
| One component | 23.291 | 0.132 | 0.09 | 0.17 |
| Two components | 22.841 | -0.318 | (-0.40 | ,-0.24) |
| Three components | 22.719 | -0.440 | (-0.54 | ,-0.34) |
| Four components | 21.484 | -1.675 | (-1.90 | ,-1.45) |
| Five components | 18.764 | -4.394 | (-4.91 | ,-3.88) |
| Increase by one component |  |  |  |  |
|  |  |  |  |  |
| CKM | RMST | RMST difference | (95% CI) | |
| Stage 0 | 23.914 |  |  |  |
| Stage 1 | 23.847 | -0.068 | (-0.08 | ,-0.05) |
| Stage 2 | 23.811 | -0.104 | (-0.12 | ,-0.09) |
| Stage 3 | 22.549 | -1.365 | (-1.48 | ,-1.25) |
| Stage 4 | 20.449 | -3.465 | (-3.69 | ,-3.24) |
| All CKM |  |  |  |  |
|  |  |  |  |  |
| Zero components | 23.808 |  |  |  |
| One component | 23.841 | 0.033 | 0.02 | 0.05 |
| Two components | 23.656 | -0.152 | (-0.18 | ,-0.12) |
| Three components | 23.553 | -0.255 | (-0.30 | ,-0.21) |
| Four components | 22.810 | -0.998 | (-1.11 | ,-0.89) |
| Five components | 21.045 | -2.763 | (-3.06 | ,-2.47) |

We additionally performed RMST regression for each CKM stage. The results showed that as CKM stage and the number of CKM components increased, RMST values progressively decreased.

**Page 10, Line 260**

“To further assess survival differences, we analyzed RMST. CKM Stage 0 serves as the reference, with an RMST of 23.395 years. CKM stage 1 showed a slight reduction in RMST (23.248 years; difference: -0.147, 95% CI: -0.19, -0.11), while CKM Stage 2 had a more pronounced decline (23.140 years; difference: -0.255, 95% CI: -0.29, -0.22). The reduction was more substantial in CKM stage 3 (21.161 years; difference: -2.234, 95% CI: -2.44, -2.03) and stage 4 (19.043 years; difference: -4.352, 95% CI: -4.72, -3.98). These results indicate a progressive decline in survival as CKM stage advances (Table S2).”

12. When making statements about significance (e.g., "However, there was no significant difference in the risk of other causes of death between CKM Stage 1 and Stage 0 participants.") then appropriate statistics should be presented to justify this claim such as a p-value for the statistical contrast testing this specific assertion.

***Response:***

We have revised this part.

**Page 10, Line 251**

“However, there was no significant difference in the risk of all-cause (HR: 0.96; 95% CI: 0.91–1.02) and CVD death (HR: 1.13; 95% CI: 0.95–1.35) between CKM Stage 1 and Stage 0 participants.”

13. "We examined the risk of death by comparing the remaining years of life expectancy in male and female participants." How was life expectancy estimated? This appears to be related to "Chiang's life table method" but reference 28 did not have sufficient detail about how this method is defined and used, rather it appeared to be another application of the method. Please ensure the techniques used and assumptions made supporting these analyses are fully detailed in the Methods so they can be replicated. This is particularly important when using Cox regression because Cox PH makes no assumptions about baseline hazard.

***Response:***

We acknowledge that CKM stage may change over time. Currently, our estimation of life expectancy is based on the follow-up person-years between the baseline CKM status in this cohort and mortality. By examining differences in life expectancy, we aim to reflect the varying mortality risks associated with different CKM stages. Regarding life expectancy, we have revised the cited references and clarified the methodology in this section.

**Page 9, Line 201**

“Life expectancy is calculated using life tables, which estimate the expected remaining years of life for individuals at a given age based on mortality rates. We estimated age-specific mortality rates based on different CKM stages and sex-specific stratifications to determine the probability of survival. Chiang’s life table method was employed to calculate years of life lost for each CKM stages and components. In this study, life expectancy refers to the average expected remaining years of life for individuals at ages 30, 40, 50, 60, and 70. Previously, we used our cohort data to estimate life expectancy based on at least 30 different health risk factors. [22, 23]”

14. I'd encourage the authors to provide their SAS code as a supplement, particularly for the more novel techniques like estimating and comparing life expectancy/years of life lost.

***Response:***

The SAS codes were provided in the supplement.

15. "Among participants with all five components, 44.4% had at least two components, 22.9% had at least three components, and 7.8% had at least four components." My apologies but I do not understand this sentence at all and am struggling with this Results paragraph more generally. Suggest rewording here and in the Discussion.

***Response:***

We have revised our sentences on this section.

**Page 11, 2^nd^ paragraph, Line 282**

“In our study cohort, one-fifth (22.7%) of the participants had at least two CKM components. However, among those with at least one CKM component, two-fifths (44.4%) had at least two components. This indicates that among individuals with at least one CKM component, the prevalence of multimorbidity (having at least two components) is relatively high. Similarly, one-ninth (11.7%) of participants in the entire cohort had at least three CKM components. However, among those with at least one CKM component, the proportion with at least three components increased to one-fifths (22.9%).”

16. [MINOR] "We examined the risk of individual CKM components (Table 3)." I think the results in this para are from Table S5-1, not Table 3?

***Response:***

We have revised accordingly. **Page 11, Line 294**

“We examined the risk of individual CKM components (Table 3, Table S6-1 and S6-2).”

17. [MINOR] "Although the relative hazard ratios of different chronic disease risks for mortality varied across different causes of death, we found that with an increasing number of components, the risk of death also increased. This finding demonstrates a dose-response relationship across all-causes of death and the incidence of ESKD." Consider putting sentences like this that interpret the results in the Discussion rather than the Results.

***Response:***

We have removed the interpretative statements from the Results section.

18. "We assessed participants' awareness of their chronic conditions based on whether they had sought medical treatment or received medication." This needs to be covered fully in the Methods before results are presented, noting the results presented are quite alarming! I think it's also worth being clear in the Introduction why questions of participant awareness are important to be considered as part of this particular study.

***Response:***

“Awareness of the chronic conditions was defined as those who had each of the CKM components who answered yes to this question.” **Page 8, Line 163.**

We have included this in the method section.

19. The authors use of the word "clustering" (e.g., "Our results revealed that CKM component clustering is positively associated with the risks of CVD mortality, all-cause mortality, diabetes mortality, and ESKD…") is a bit confusing because clustering has specific meanings in statistics and data science. Consider replacing with more precise language like "the risk of mortality increased with the number of CKM components observed" rather than using "clustering" as shorthand.

***Response:***

We have so revised. **Page 13, Line 353**

“Our results revealed that the risk of mortality increased with the number of CKM components observed. Although different risks contribute to various causes of death at varying levels, increased number of components was consistently correlated with a higher risk of mortality.”

20. PAF results are introduced for the first time in the Discussion. Please describe how PAF was calculated in the Methods and present results in the Results section before discussing in the Discussion section.

***Response:***

We have described the PAF in the Methods.

**Page 9, Line 209.**

“The population-attributable fraction of CKM was defined as P×(HR–1)/(1+P×[HR–1]) where P is the prevalence of CKM components and HR is the hazard ratio (for all-cause mortality).” The total PAF of CKM was estimated by the following formula

$\sum_{i=1}^{n} Pi(RRi-1)$ / $\sum_{i=1}^{n} Pi(RRi-1)+1$

where *i* signifies the stages of CKM (i = 1,…, n).

21. "Such individuals likely represent a healthier and socioeconomically advantaged segment of the population, thereby limiting the generalizability of our findings to the broader population. However, the use of relative risks (HRs) in our analysis helps mitigate this limitation by allowing for comparisons within groups." Hazard ratios (or any other statistic) do not assist with generalizability of results if the sample itself is biased as described. Also, hazard ratios are most definitely not relative risks. Please reword.

***Response:***

We agreed with this point that “hazard ratios do not assist with generalizability of results if the sample itself is biased as described.

We have deleted this sentence.

**Page 15, Line 431**

Second, we recruited participants who were undergoing health check-ups. Such individuals likely represent a healthier and socioeconomically advantaged segment of the population, thereby limiting the generalizability of our findings to the broader population. ~~However, the use of relative risks (HRs) in our analysis helps mitigate this limitation by allowing for comparisons within groups.~~

22. "Third, our analysis was based only on baseline measurements, which do not account for changes in individual circumstances over time." Ah! This answers my earlier questions. This is a very serious limitation. This should have been made clearer much earlier in the Methods and justification provided as to the validity of the findings in the face of this limitation.

***Response:***

We have revised accordingly in the Methods.

**Page 7, 2^nd^ paragraph, Line 126**

“Participants were non-institutionalized, generally healthy individuals from diverse backgrounds. They were encouraged to undergo annual health screenings, during which identical questionnaires were administered at each visit. However, for this study, only baseline data from the initial visit were used for analysis.”

23. "However, sensitivity analyses using data from participants who underwent two examinations yielded similar results (Table S8)." I do not think this analysis mitigates the limitation identified. All CKM components identified (hypertension, CKD, diabetes, metabolic syndrome, and hyperlipidaemia) emerge and change over time, and this ostensibly simple analysis does not mitigate this clinical reality. Also, again, these results presented in the Discussion were not previously included in the Methods or Results.

***Response:***

We have added this in the Methods. **Page 9, Line 215**

“We conducted an analysis among participants who had undergone two health examinations, using their second CKM stage assessment to evaluate differences in mortality risk. Additionally, we have incorporated a time-dependent analysis for participants with a second examination as a sensitivity analysis. This approach accounts for changes in CKM stages over time and the duration of follow-up, allowing us to further evaluate the association between CKM stages and mortality outcomes.”

Furthermore, in the study limitations, we have suggested that future research should focus on the impact of CKM stage changes over time to provide a more comprehensive understanding of its implications.

**Page 16, Line 435**

“Third, our analysis was based only on baseline measurements, which do not account for changes in individual circumstances over time. Future research should focus on the impact of CKM stage changes over time to provide a more comprehensive understanding of its implications. However, we conducted an analysis among participants who had undergone two health examinations, using their second CKM stage assessment to evaluate differences in mortality risk. We also incorporated a time-dependent analysis, and this yielded similar results (Table S12).”

Reviewer #2: The paper entitled: "Unveiling the Creation of Cardiovascular-Kidney-Metabolic Syndrome: the Contribution to All-Cause and Cardiovascular Mortality - A cohort of more than half-a-million participants" was aimed to clarify the role of cardiovascular-kidney-metabolic (CKM) syndrome as risk factor for the of all-cause and cardiovascular mortality, and end-stage kidney disease (ESKD). For this purpose, the authors analyzed a cohort of 515,602 individuals that were followed for almost 25 years. The CKM syndrome was documented in 71.5% of the study population, the age increased the prevalence of the CKM syndrome. CKM syndrome increased the risk of all-cause and cardiovascular mortality, and ESKD. Basically, this is an interesting analysis, with an important epidemiological implication about cardiovascular risk. Discussion is focused on the topic of the paper with a correct description of the strengths and limitations of the study. However, the paper presents few

weaknesses. Therefore, I invite the authors to address the criticisms that I raised:

Major comments

1) The classification of the severity of the CKM syndrome should be consistent with to that reported by the AHA, mentioned in the reference #19.

***Response:***

The classification of the CKM syndrome is consistent with that by the AHA. We have added a new table for CKM syndrome definition used in this study (Table S1).

| **CKM Syndrome Stages** | **Definition** |  |
| --- | --- | --- |
| Stage 0: No CKM risk factors | Individuals with normal BMI and waist circumference, normoglycemia, normotension, a normal lipid profile, and no evidence of CKD or subclinical or clinical CVD |  |
| Stage 1: Excess or dysfunctional adiposity | Individuals with overweight/obesity, abdominal obesity, or dysfunctional adipose tissue, without the presence of other metabolic risk factors or CKD BMI^a^ ≥ 23 kg/m^2^,  waist circumference^a^ ≥ 80 cm for women and ≥ 90 cm for men, or  fasting blood glucose level = 100–124 mg/dL.^b^ |  |
| Stage 2: Metabolic risk factors and CKD | Presence of hypertension,^c^  diabetes,^d^  metabolic syndrome,^e^  high triglyceride levels (≥ 135 mg/dL),^f^ or  any stage of CKD.^g^ |  |
| Stage 3: Subclinical CVD in CKM | With a Predicting Risk of cardiovascular disease EVENTs (PREVENT) score indicating a 10-year risk of CVD exceeding 20% or  has a kidney function with an eGFR of ≤30 mL/min/1.73 m². |  |
| Stage 4: Clinical CVD in CKM | Self-reports medication use or seeks medical attention for coronary heart disease, heart failure, or stroke or  has CKD with an eGFR of ≤30 mL/min/1.73 m^2^. |  |
| ^a^ Asian criteria  ^b^ HbA1c are not available in this cohort  ^c^ Hypertension was defined as a history of hypertension, the use of antihypertensive medication, or a systolic blood pressure of ≥130 mmHg  or a diastolic blood pressure of ≥80 mmHg.  ^d^ Diabetes was defined as a history of diabetes, the use of diabetes medication, or a fasting blood glucose level of ≥126 mg/dL.  ^e^ Metabolic syndrome was defined as the presence of at least three of the following criteria: waist circumference≥80 cm for women and ≥90 cm for men;  systolic blood pressure ≥130 mmHg or diastolic blood pressure ≥80 mmHg and/or use of antihypertensive medications;  fasting blood glucose ≥100 mg/dL;  HDL cholesterol <40 mg/dL for men and <50 mg/dL for women;  triglycerides ≥150 mg/dL.  ^f^ High triglyceride levels was defined as a history of hyperlipidemia, the use of lipid-lowering medication, or a triglyceride level of ≥135 mg/dL.  ^g^ CKD was determined on the basis of eGFR by using the CKD Epidemiology Collaboration (CKD-EPI) formula,  and proteinuria levels were detected using a dipstick test, categorized as negative, trace, 1+, 2+, or 3+.  ^h^ Calculated by the equation of PREVENT exceeding 20% risk | | |

2) The Authors should report in all tables and figures how are presented the results (mean ± SD, percentages ….).

***Response:***

We have presented in all tables and figures.

**Page 10, 1^st^ paragraph, Line 225**

“The study cohort included 515,602 participants, with a mean (standard deviation) age of 40.3 (13.4) years at recruitment. Among them, 257,535 (49.9%) were women. The majority of participants (n= 368,578 participants, (71.5%)) met criteria for CKM syndrome, with 19.5%, 46.3%, 1.9%, and 3.8% classified as CKM stages 1, 2, 3, and 4, respectively. Distribution of sociodemographic characteristics stratified by CKM stages were shown in Table 1. The prevalence of CKM syndrome increased with age, exceeding 90% among participants aged 55 years or older, with CKM stages 3–4 becoming more prevalent in this age group (Figure 1 and Figure S4). Participants with CKM syndrome were more likely to be male (58.77%), have an educational level of middle school or lower (27.5%), current smoker (25.2%), and consume alcohol regularly (10.6%) Additionally, physical inactivity was more common among those with CKM syndromes, with 48.9% (170,635 participants) reporting physically inactive.”

Minor comments:

1) The title should be simplified. I think that "The Cardiovascular-Kidney-Metabolic Syndrome: the Contribution to All-Cause and Cardiovascular Mortality" is enough.

***Response:***

We have revised our title: “The Cardiovascular-Kidney- Metabolic Syndrome: the Contribution to All-Cause and Cardiovascular Mortality”.

2) The English should be accurately checked.

***Response:***

We have again checked our manuscript by native speakers.

3) The introduction can be shortened. History of CKM syndrome can be erased.

***Response:***

We agreed with this point. We have shortened our introduction and erased the history of CKM syndrome.

**Introduction, 2^nd^ paragraph, Line 77:**

“In November 2023, the American Heart Association (AHA) published new guidelines that emphasize the importance of cardiovascular–kidney–metabolic (CKM) health for the first time [8, 9]. Patients with CVD often have comorbid hypertension, kidney disease, and metabolic disorders, which share common underlying mechanisms and have a critical impact on CVD incidence and mortality [10-13]. This CKM syndrome is defined and staged based on the presence of clinical characteristics (e.g., obesity) and comorbidities (e.g., hypertension, diabetes, and chronic kidney disease (CKD)). Because CKM syndrome is a newly defined concept, it is essential to understand its impact on mortality risk and CVD outcomes, as well as the distribution of different CKM stages in the general population. Additionally, CKM introduces an interdisciplinary approach to health, shifting the focus from individual disease factors to the interactions among multiple diseases. CKM syndrome emphasize the importance of multidisciplinary approaches to prevention, risk stratification, and treatment for these diseases. Unlike previous models for CVD risk prediction, CKM syndrome incorporates kidney disease-related risk factors. Recently, international scientific societies have advocated for the inclusion of kidney disease in the World Health Organization’s (WHO) framework on major non-communicable disease (NCD) determinants of premature mortality [14]. However, while incorporating CKD into this framework acknowledges its significance, concerns remain regarding its predictive utility for all-cause and CVD outcomes, particularly given the limited public awareness of CKD[15]. Recent therapeutic advances further support the rationale for an integrated CKM framework, as several novel medications demonstrate beneficial effects across multiple disease domains [16]. Sodium-glucose cotransporter-2 (SGLT2) inhibitors, for example, have shown remarkable efficacy in reducing cardiovascular events, slowing CKD progression, and improving glycemic control [17]. Similarly, glucagon-like peptide-1 (GLP-1) receptor agonists [18] and nonsteroidal mineralocorticoid receptor antagonists (MRAs) exhibit protective effects across multiple organ systems [19]. These findings suggest that optimal treatment strategies should consider the entire CKM spectrum rather than focusing on isolated conditions.”

Reviewer #3: 1. The authors only mentioned that about 0.5 million participants were included from a health screening program. However, it is not clear who the participants were or how they were selected. Was participation in health screening program voluntary or mandatory? Were there specific inclusion and exclusion criteria? How well do the participants represent the general population or the target population of interest? What are the scope and purpose of the health screening program, and have these factors influenced the study population toward a healthier or less healthy group? Without this information, it is unclear whether there is potential bias in the study.

***Response:***

We agreed with reviewer’s suggestion. We have revised our purpose of this study in the introduction and have added a detailed description of our study cohort in the Methods section.

**Introduction, 2^nd^ paragraph, Line 77:**

“In November 2023, the American Heart Association (AHA) published new guidelines that emphasize the importance of cardiovascular–kidney–metabolic (CKM) health for the first time [8, 9]. Patients with CVD often have comorbid hypertension, kidney disease, and metabolic disorders, which share common underlying mechanisms and have a critical impact on CVD incidence and mortality [10-13]. This CKM syndrome is defined and staged based on the presence of clinical characteristics (e.g., obesity) and comorbidities (e.g., hypertension, diabetes, and chronic kidney disease (CKD)). Because CKM syndrome is a newly defined concept, it is essential to understand its impact on mortality risk and CVD outcomes, as well as the distribution of different CKM stages in the general population. Additionally, CKM introduces an interdisciplinary approach to health, shifting the focus from individual disease factors to the interactions among multiple diseases. CKM syndrome emphasize the importance of multidisciplinary approaches to prevention, risk stratification, and treatment for these diseases. Unlike previous models for CVD risk prediction, CKM syndrome incorporates kidney disease-related risk factors. Recently, international scientific societies have advocated for the inclusion of kidney disease in the World Health Organization’s (WHO) framework on major non-communicable disease (NCD) determinants of premature mortality [14]. However, while incorporating CKD into this framework acknowledges its significance, concerns remain regarding its predictive utility for all-cause and CVD outcomes, particularly given the limited public awareness of CKD[15]. Recent therapeutic advances further support the rationale for an integrated CKM framework, as several novel medications demonstrate beneficial effects across multiple disease domains [16]. Sodium-glucose cotransporter-2 (SGLT2) inhibitors, for example, have shown remarkable efficacy in reducing cardiovascular events, slowing CKD progression, and improving glycemic control [17]. Similarly, glucagon-like peptide-1 (GLP-1) receptor agonists [18] and nonsteroidal mineralocorticoid receptor antagonists (MRAs) exhibit protective effects across multiple organ systems [19]. These findings suggest that optimal treatment strategies should consider the entire CKM spectrum rather than focusing on isolated conditions.

Therefore, this study investigates the combinations of different CKM components and their association with mortality risk, including hypertension, chronic kidney disease, diabetes mellitus, metabolic syndrome, and hyperlipidemia. Previously, we used a large cohort to examine the prevalence and mortality burden of CKD [20]. We also accessed the public awareness of each of the CKM components. In the present study, we investigated the risks of all-cause mortality, CVD mortality, and ESKD associated with various CKM components. Additionally, we examined the effects of individual and multiple CKM components on mortality and ESKD risk.”

2. The prevalence of CKM is unexpectedly high. Even in the youngest age group of 20-24 years, nearly 50% of individuals had prevalent CKM at baseline. Could the authors provide any explanations? Is this prevalence comparable to those reported in other studies?

***Response:***

Thank you to the reviewer for the careful observations.

Indeed, the prevalence of CKM is unexpectedly high. However, since our cohort is relatively younger (with a mean age of 40 years at baseline), the overall CKM prevalence in our study is relatively lower than that reported in the U.S. NHANES dataset. Additionally, our definition of CKM Stage 1 was adapted for an Asian population, which also influences the CKM prevalence observed in our study.

At ages 20–24 years, it is true that nearly 50% (47.3%) of individuals had prevalent CKM at baseline. This is primarily due to:

CKM Stage 1: 18% of participants had a BMI ≥ 23, which is the cutoff used for this population. CKM Stage 2: Approximately 20% had blood pressure ≥ 130/80 mmHg and a certain proportion had high triglyceride levels (≥ 135 mg/dL), leading to an overall CKM Stage 2 prevalence of approximately 28% in this age group.

To further assess the comparability of our findings, we compared CKM prevalence between our cohort and NHANES for participants aged 20–44 years. The estimated prevalence of CKM stages in each dataset is as follows:

In our cohort: Stage 1: 22.0%; Stage 2: 39.0%; Stage 3: 0.02%; Stage 4: 1.3%.

In NHANES: Stage 1: 37.6%; Stage 2: 43.2%; Stage 3: 0.2%; Stage 4: 1.7%

For CKM Stages 3 and 4, our cohort and NHANES show similar prevalence rates in a comparable age group. However, CKM Stage 1 in NHANES is primarily defined by obesity, while Stage 2 prevalence is influenced by the distribution of individuals with blood pressure ≥ 130/80 mmHg.

Therefore, we believe that our CKM prevalence estimates are comparable to those reported in other studies.

3. The study population is very young, with a mean age of 40 years at baseline. How might this affect the generalisability of the study findings to the general population?

***Response:***

We agree with the reviewer’s comments.

Our cohort is indeed relatively younger, which may affect the generalizability of the prevalence estimates. To account for this impact, we have added new age and education-adjusted prevalence estimates to better project the prevalence among this Asian-majority population.

**Page 15, Line 431**

We have also addressed this in the limitation:

“Second, we recruited participants who were undergoing health check-ups. Such individuals likely represent a healthier and socioeconomically advantaged segment of the population, thereby limiting the generalizability of our findings to the broader population. In addition, our cohort is relatively younger mean (SD) age, 40.3 years (13.4), which may affect the generalizability of the prevalence estimates.”

4. The definition of CKM uses several laboratory measurements, and individuals with missing data were excluded from the analysis. How do these missing data affect the prevalence of CKM and its association with mortality? Have the authors considered using other methods to handle the missing data?

***Response:***

Some participants at earlier stages operated did not receive questionnaires, and some data were therefore missing for specific elements. In this cohort, we used data for those with complete questionnaires data.

We added a new table for multiple imputed data to account for missing baseline information and outcomes.

**Page 11, Line 267**

“The robustness of the findings was assessed using multiple imputed data to account for missing baseline information and outcomes (Table S3). The results for those with multiple imputation and those excluded due to missing data were generally similar.”

5. A description of baseline characteristics of participants is needed in the result section.

***Response:***

We have added the baseline characteristics of participants in results.

**Page 10, Results**

“The study cohort included 515,602 participants, with a mean (standard deviation) age of 40.3 (13.4) years at recruitment. Among them, 257,535 (49.9%) were women. The majority of participants (n= 368,578 participants, (71.5%)) met criteria for CKM syndrome, with 19.5%, 46.3%, 1.9%, and 3.8% classified as CKM stages 1, 2, 3, and 4, respectively. Distribution of sociodemographic characteristics stratified by CKM stages were shown in Table 1. The prevalence of CKM syndrome increased with age, exceeding 90% among participants aged 55 years or older, with CKM stages 3–4 becoming more prevalent in this age group (Figure 1 and Figure S4). Participants with CKM syndrome were more likely to be male (58.77%), have an educational level of middle school or lower (27.5%), current smoker (25.2%), and consume alcohol regularly (10.6%) Additionally, physical inactivity was more common among those with CKM syndromes, with 48.9% (170,635 participants) reporting physically inactive.”

6. Whether other severe medical conditions, such as cancer or other chronic diseases, were considered as confounders or excluded from the analysis?

***Response:***

Participants who self-reported the use of CKM conditions were included in the CKM definitions. For example, self-reported antihypertensive medication or a history of hypertension were classified under the hypertension component.

For other chronic diseases like cancer history, we have added cancer information as covariates in the sensitivity analysis.

**Page 11, Line 270**

“Additionally, we conducted a sensitivity analysis considering the use of analgesics and cancer history. After incorporating the variables, the main results, including all-cause mortality and cardiovascular mortality, remained unchanged.”

General editorial requests:
(Note: not all will apply to your paper, but please check each item carefully)

*       We ask every co-author listed on the manuscript to fill in a contributing author statement, making sure to declare all competing interests. If any of the co-authors have not filled in the statement, we will remind them to do so when the paper is revised. If all statements are not completed in a timely fashion this could hold up the re-review process. If new competing interests are declared later in the revision process, this may also hold up the submission. Should there be a problem getting one of your co-authors to fill in a statement we will be in contact. Please do not add or remove authors without first discussing this with the handling editor. You can see our competing interests policy here: <http://journals.plos.org/plosmedicine/s/competing-interests>.
***Response:***

We have disclosed all competing interests in accordance with PLOS Medicine’s regulations.

Page 17

Competing Interests Statement

The authors have declared that no competing interests exist.

We have also provided the PLOS Medicine office with a confirmation letter signed by all authors.

*       Please upload any figures associated with your paper as individual TIF or EPS files with 300dpi resolution at resubmission; please read our figure guidelines for more information on our requirements: <http://journals.plos.org/plosmedicine/s/figures>. While revising your submission, please upload your figure files to the PACE digital diagnostic tool, <https://pacev2.apexcovantage.com/>. PACE helps ensure that figures meet PLOS requirements. To use PACE, you must first register as a user. Then, login and navigate to the UPLOAD tab, where you will find detailed instructions on how to use the tool. If you encounter any issues or have any questions when using PACE, please email us at [PLOSMedicine@plos.org](mailto:PLOSMedicine@plos.org).
***Response:***

We have edited all the figures using PACE and re-uploaded the revised versions.

*       Please ensure that the paper adheres to the PLOS Data Availability Policy (see <http://journals.plos.org/plosmedicine/s/data-availability>), which requires that all data underlying the study's findings be provided in a repository or as Supporting Information. For data residing with a third party, authors are required to provide instructions with contact information (web or email address) for obtaining the data. Please note that a study author cannot be the contact person for the data. PLOS journals do not allow statements supported by "data not shown" or "unpublished results." For such statements, authors must provide supporting data or cite public sources that include it.
***Response:***

We have provided the Data Availability statement in accordance with the PLOS Data Availability Policy.

Page 17

**Data sharing statement**

The data used in this research were authorized by MJ Health Research Foundation (Authorization Code: MJHRFB2014001C). The MJ Health Research Foundation administered the MJ Health Survey Database and MJ BioData, and the data are available at http://www.mjhrf.org.

*       We expect all researchers with submissions to PLOS in which author-generated code underpins the findings in the manuscript to make all author-generated code available without restrictions upon publication of the work. In cases where code is central to the manuscript, we may require the code to be made available as a condition of publication. Authors are responsible for ensuring that the code is reusable and well documented. Please make any custom code available, either as part of your data deposition or as a supplementary file. Please add a sentence to your data availability statement regarding any code used in the study, e.g. "The code used in the analysis is available from Github [URL] and archived in Zenodo [DOI link]" Please review our guidelines at <https://journals.plos.org/plosmedicine/s/materials-software-and-code-sharing> and ensure that your code is shared in a way that follows best practice and facilitates reproducibility and reuse. Because Github depositions can
be readily changed or deleted, we encourage you to make a permanent DOI'd copy (e.g. in Zenodo) and provide the URL.

***Response:***

We have provided the Code availability statement as follows.

**Code availability**

Code used for study analyses may be made available upon reasonable request to the authors.

*       FINANCIAL DISCLOSURES: The funding statement should include: specific grant numbers, initials of authors who received each award, URLs to sponsors’ websites. Also, please state whether any sponsors or funders (other than the named authors) played any role in study design, data collection and analysis, the decision to publish, or preparation of the manuscript. If they had no role in the research, include this sentence: “The funders had no role in study design, data collection and analysis, decision to publish, or preparation of the manuscript.”
***Response:***

We have included this sentence as follows.

**Funding**

Mr. Min-Kuang Tsai received grants from National Science and Technology Council (NSTC 113-2314-B-038-024) and Taipei Medical University-Shuang Ho Hospital (113FRP-20). This study is supported in part by the Taiwan Ministry of Health and Welfare Clinical Trial Center (MOHW110-TDU-B-212-124004). The funders had no role in study design, data collection and analysis, decision to publish, or preparation of the manuscript.

*       COMPETING INTEREST: All authors must declare their relevant competing interests per the PLOS policy, which can be seen here: <https://journals.plos.org/plosmedicine/s/competing-interests>
For authors with ties to industry, please indicate whether any of the interests has a financial stake in the results of the current study.

***Response:***

We have included this as follows.

**Competing interests statement**

The authors have declared that no competing interests exist.

*       ETHICS STATEMENTS: Please provide the approval number and details on the consent procedure (e.g., oral or waived by the ethics committee).
***Response:***

We have included Ethics statements as follows.

**Ethics statements**

This study was approved by the Institutional Review Board of Taipei Medical University (Approval Number: N202404037). All participants gave written consent to analysis their unidentifiable data before being included in this study.

FORMATTING - GENERAL
*       Abstract: Please structure your abstract using the PLOS Medicine headings (Background, Methods and Findings, Conclusions). Please combine the Methods and Findings sections into one section.

***Response:***
We have revised the abstract accordingly.

*       At this stage, we ask that you include a short, non-technical Author Summary of your research to make findings accessible to a wide audience that includes both scientists and non-scientists. The Author Summary should immediately follow the Abstract in your revised manuscript. This text is subject to editorial change and should be distinct from the scientific abstract. Ideally each sub-heading should contain 2-3 single sentence, concise bullet points containing the most salient points from your study. In the final bullet point of 'What Do These Findings Mean?', please include the main limitations of the study in non-technical language. Please see our author guidelines for more information: <https://journals.plos.org/plosmedicine/s/revising-your-manuscript#loc-author-summary>.
***Response:***

Author Summary has been included.

### Why was this study done?

- In November 2023, the American Heart Association (AHA) introduced new guidelines emphasizing the importance of cardiovascular–kidney–metabolic (CKM) health for the first time.
- As CKM syndrome is a newly defined concept, understanding its impact on mortality risk and cardio-vascular disease (CVD) outcomes, as well as the distribution of CKM stages in the general population is crucial.

### What did the researchers do and find?

- We investigated the epidemiological distribution of CKM syndrome and its associations with mortality outcomes in a large Asian cohort.
- CKM syndrome was prevalent in 71.5% of participants, with nearly 90% of those aged ≥55 years affected. Among all participants, one-fifth (22.7%) of the participants had at least two components, while one-ninth (11.7%) had at least three.
- We found that the risks of all-cause mortality, CVD mortality, and incident end-stage kidney disease (ESKD) increased with the number of CKM components present.

### What do these findings mean?

- CKM syndrome should not be treated as a set of isolated conditions. Physicians should adopt an **interdisciplinary approach**, addressing all five components when any single disease component is diagnosed to ensure **comprehensive, cross-specialty care**.
- The primary limitation of this study is that our analysis relied on baseline measurements only, without accounting for longitudinal changes. Future research should explore how CKM stage transitions over time influence health outcomes for a more comprehensive understanding of its implications.

*       Please express the main results with 95% CIs as well as p values. When reporting p values please report as p<0.001 and where higher as the exact p value p=0.002, for example. Throughout, suggest reporting statistical information as follows to improve clarity for the reader "22% (95% CI [13%,28%]; p</=)". Please be sure to define all numerical values at first use.
***Response:***

All results with 95% CIs were included.

*       Please include page numbers and line numbers in the manuscript file. Use continuous line numbers (do not restart the numbering on each page).

***Response:***

Page numbers and line number have been included.

*       Please cite the reference numbers in square brackets. Citations should precede punctuation.
***Response:***

We have revised the reference numbers accordingly.

FIGURES AND TABLES
*       Please provide titles and legends for all figures and tables (including those in Supporting Information files).
***Response:***

Titles and figure legends have been provided.

*       Please define all abbreviations used in each figure/table (including those in Supporting Information files).
***Response:***

We have define all abbreviations in each table/figure.

*       Please consider avoiding the use of red and green in order to make your figure more accessible to those with color blindness.

***Response:***

We have revised our figures accordingly.

SUPPLEMENTARY MATERIAL
*       Please note that supplementary material will be posted as supplied by the authors. Therefore, please amend it according to the relevant comments outlined here.
***Response:***

Supplementary tables and figures have been revised.

*       Please cite your Supporting Information as outlined here: <https://journals.plos.org/plosmedicine/s/supporting-information>
***Response:***

The citation for supplementary tables and figures have been revised.

REFERENCES
*       PLOS uses the numbered citation (citation-sequence) method and first six authors, et al.
***Response:***

We have revised accordingly.

*       Please ensure that journal name abbreviations match those found in the National Center for Biotechnology Information (NCBI) databases (<http://www.ncbi.nlm.nih.gov/nlmcatalog/journals>), and are appropriately formatted and capitalised.

***Response:***

The journal name abbreviations have been matched with NCBI databases.

*       Where website addresses are cited, please include the complete URL and specify the date of access (e.g. [accessed: 12/06/2024]).

***Response:***

We did not cite website reference.

*       Please also see <https://journals.plos.org/plosmedicine/s/submission-guidelines#loc-references> for further details on reference formatting.

***Response:***

We have revised accordingly.

STUDY TYPE-SPECIFIC REQUESTS

*       Abstract: Please include the study design, population and setting, number of participants, years during which the study took place (enrollment and follow up), length of follow up, and main outcome measures.
***Response:***

We have revised accordingly.

“Background

The American Heart Association recently issued guidelines introducing the concept of cardiovascular–kidney–metabolic (CKM) syndrome to emphasize the importance of multidisciplinary approaches to prevention, risk stratification, and treatment for these diseases. This study assessed the prevalence of CKM syndrome stages and the mortality risk associated with its components in a large Asian cohort.

Methods and Findings

We analyzed a cohort of 515,602 participants aged ≥20 years from a health screening program conducted between 1996 and 2017. We assessed the associations of all-cause mortality, cardio-vascular disease (CVD) mortality, and cause-specific mortality with CKM stages and its components—hypertension, diabetes mellitus (DM), chronic kidney disease (CKD), metabolic syndrome, and hyperlipidemia. Multivariate Cox proportional hazards models were used to calculate hazard ratios (HRs). We used Chiang’s life table method to estimate years of life lost due to each CKM component. In this cohort, 71.5% of the participants met criteria for CKM syndrome, with prevalence rates of 19.5%, 46.3%, 1.9%, and 3.8% for stages 1, 2, 3, and 4, respectively. CKM syndrome was associated with higher risks of all-cause mortality (HR: 1.33; 95% confidence interval, CI: 1.28, 1.39), CVD mortality (HR: 2.81; 95% CI: 2.45, 3.22) and incident end-stage kidney disease (ESKD) (HR: 10.15; 95% CI: 7.54, 13.67). Each additional CKM component was associated with a 22% increase in the risk of all-cause mortality (HR: 1.22; 95% CI: 1.21, 1.23), a 37% increase in the risk of CVD mortality (HR: 1.37; 95% CI: 1.35, 1.40) compared with those without any CKM components. In addition, each additional component reduced average life expectancy by 3 years. The population attributable fractions of CKM syndrome were 18.7% (95% CI: 15.8, 21.7) for all-cause mortality and 55.0% (95% CI: 49.0, 60.4) for CVD mortality. We estimated that failing to include CKD in CKM syndrome could result in the missed attribution of 11% of CVD deaths.

Conclusions

In the large longitudinal study, the prevalence of CKM syndrome and its components were associated with dose-response risks of all-cause mortality, CVD mortality, and ESKD. These findings highlight the clinical need for integrated care within CKM health.”

*       Please ensure that the study is reported according to the STROBE (or appropriate STOBE extension) guideline (available from: <https://www.equator-network.org/reporting-guidelines/strobe>) and include the completed STROBE (or STROBE extension) checklist as Supporting Information. Please add the following statement, or similar, to the Methods: "This study is reported as per the Strengthening the Reporting of Observational Studies in Epidemiology (STROBE) guideline (S1 Checklist)." When completing the checklist, please use section and paragraph numbers, rather than page numbers.

***Response:***
The STROBE checklist has been provided.

*       For all observational studies, in the manuscript text, please indicate: (1) the specific hypotheses you intended to test, (2) the analytical methods by which you planned to test them, (3) the analyses you actually performed, and (4) when reported analyses differ from those that were planned, transparent explanations for differences that affect the reliability of the study's results. If a reported analysis was performed based on an interesting but unanticipated pattern in the data, please be clear that the analysis was data driven.
***Response:***

We have stated our research objectives and hypotheses in the Introduction.

“Therefore, this study investigates the combinations of different CKM components and their association with mortality risk, including hypertension, chronic kidney disease, diabetes mellitus, metabolic syndrome, and hyperlipidemia. Previously, we used a large cohort to examine the prevalence and mortality burden of CKD [20]. We also accessed the public awareness of each of the CKM components. In the present study, we investigated the risks of all-cause mortality, CVD mortality, and ESKD associated with various CKM components. Additionally, we examined the effects of individual and multiple CKM components on mortality and ESKD risk.”

Additionally, we have detailed our research methods and statistical approaches in the Methods section.

*       Please state in the Methods section whether the study had a prospective protocol or analysis plan. If a prospective analysis plan (from your funding proposal, IRB or other ethics committee submission, study protocol, or other planning document written before analyzing the data) was used in designing the study, please include the relevant document(s) with your revised manuscript as a Supporting Information file to be published alongside your study and cite it in the Methods section. A legend for this file should be included at the end of your manuscript. If no such document exists, please make sure that the Methods section transparently describes when analyses were planned, and when/why any data-driven changes to analyses took place. Changes in the analysis, including those made in response to peer review comments, should be identified as such in the Methods section of the paper, with rationale.
***Response:***

We have stated in the Methods section this study is a retrospective cohort study.

“In a retrospective cohort of 515,602 participants aged ≥20 years, medical screenings were conducted from 1996 to 2017, resulting in 8.30 million person-years of follow-up data on all-cause mortality, CVD, and ESKD over a 25-year period.”
