# Supplementary material for: Cardiovascular–kidney–metabolic syndrome and all-cause and cardiovascular mortality: A retrospective cohort study
Source: PLoS Med. 2025 Jun 26;22(6):e1004629. doi: 10.1371/journal.pmed.1004629 (PMC12200875; doi:10.1371/journal.pmed.1004629)
Supplement: Attachment — Submitted filename: Response_Reviewer_Comments_20250310.docx [file pmed.1004629.s024.docx]

Comments from the reviewers:

Reviewer #1: Thanks to the authors for the clear and cogent responses to my feedback and associated changes to the manuscript. In most cases I agree and consider the items closed (assume this is the case unless otherwise indicated). I have identified some follow-up points for further consideration that I have detailed below.

*Response:*

Thank you for the reviewers’ suggestions. Your feedback has significantly improved the quality of our study. We have conducted a detailed analysis based on your comments to further clarify our research findings, enhance the readability of our manuscript, and ensure that readers gain a better understanding of the study’s content and its limitations.

Point 6
Minor point first. I think supplementary table S12 has been mislabeled as S13, and I am assuming the content in the current "S13" pertains to this point.
Looking at the left half of the table, I don't quite understand the value of adding analyses based only on the second set of results of those patients who underwent two health examinations. There is no particular reason to believe these results would be different to the main results except for a different distribution of patients across the CKM levels, assuming patients increase CKM stages over time. I suggest this half of the table is removed or the further reasoning provided. More informative would be to report the change profile of patients with two measures. How separated in time were the first and second measures on average? Did patients tend to stay in the same CKM stage or change stages? If they changed stages, did they go up or down? By how many levels?

*Response:*

Thank you for the reviewer’s suggestions.

We have removed the left half of original Supplementary Table S12. Additionally, we have provided a new table to report the change profile of patients with two measurements.

The average follow-up time between these two examinations was 2.5 years (mean (SD): 2.5 (2.4)). Among the patients, approximately two-thirds (N = 148,639, 67.4%) remained in the same CKM stage. About 18% (N = 40,320, 18.3%) progressed to a more severe stage, while approximately 14% (N = 31,423, 14.3%) improved to a less severe stage. We conducted a Spearman correlation analysis on the CKM stages at these two time points, yielding a correlation coefficient of 0.6241 (*P* < 0.0001).

**Table S12. The distribution of baseline cardiovascular–kidney-metabolic syndrome status and follow-up CKM status among participants with a second visit**

|  | 2nd visit CKM status | | |  |  |  |  |  |  |  |  |  |
| --- | --- | --- | --- | --- | --- | --- | --- | --- | --- | --- | --- | --- |
|  | Stage 0 | | Stage 1 | | Stage 2 | | Stage 3 | | Stage 4 | | Total |  |
| Baseline CKM status | N | (%) | N | (%) | N | (%) | N | (%) | N | (%) | N | (%) |
| Stage 0 | 44,914 | (20.4) | 9,676 | (4.4) | 10,958 | (5.0) | 30 | (0.0) | 388 | (0.2) | 65,966 | (29.9) |
| Stage 1 | 6,276 | (2.8) | 23,331 | (10.6) | 14,454 | (6.6) | 47 | (0.0) | 409 | (0.2) | 44,517 | (20.2) |
| Stage 2 | 10,280 | (4.7) | 13,377 | (6.1) | 73,298 | (33.3) | 1,566 | (0.7) | 2,457 | (1.1) | 100,978 | (45.8) |
| Stage 3 | 8 | (0.0) | 10 | (0.0) | 625 | (0.3) | 1,682 | (0.8) | 335 | (0.2) | 2,660 | (1.2) |
| Stage 4 | 92 | (0.0) | 90 | (0.0) | 547 | (0.2) | 118 | (0.1) | 5,414 | (2.5) | 6,261 | (2.8) |
| Total | 61,570 | (27.9) | 46,484 | (21.1) | 99,882 | (45.3) | 3,443 | (1.6) | 9,003 | (4.1) | 220,382 | (100.0) |

Abbreviations: CKM: cardiovascular–kidney–metabolic syndrome; N: number of participants;

The average follow-up time between these two examinations was 2.5 years (mean (SD): 2.5 (2.4)).

We have included the relevant discussion in the study limitations:

**Page 17, Line 454**

“Third, our analysis was based only on baseline measurements, which do not account for changes in individual circumstances over time. Future research should focus on the impact of CKM stage changes over time to provide a more comprehensive understanding of its implications. However, we conducted an analysis among participants who had undergone two health examinations. Among them, we assessed the association between changes in CKM stages and mortality risk. The average follow-up duration between these two examinations was 2.5 years (mean (SD): 2.5 (2.4)). Approximately two-thirds (N = 148,639, 67.4%) of participants remained in the same CKM stage, while 18.3% (N = 40,320) progressed to a more severe stage, and 14.3% (N = 31,423) improved to a less severe stage (Table S12). Spearman correlation analysis of CKM stages between the two time points showed a correlation coefficient of 0.62 (*P* < 0.0001). We incorporated a time-dependent analysis, which yielded similar results (Table S13). However, given the relatively small subset of patients with repeated measurements, this additional longitudinal data point may lack sufficient power to detect true differences.”

The inclusion of time-dependent analyses as the right half of the table makes more sense and I would suggest it remains in the supplement. However, having only one extra longitudinal data point for a relatively small subset of patients is unlikely to be powered to detect any true difference, and I suggest this is briefly acknowledged as a limitation.

*Response:*

We completely agree with the reviewer’s suggestion.

We have retained the time-dependent analyses. Additionally, we have addressed this limitation by stating that this extra longitudinal data point for a relatively small subset of patients is unlikely to be sufficiently powered to detect any true differences.

I remain concerned that there is a separation of ~16.5 years between ascertainment of CKM stage and end of follow up. That said, I appreciate it is not possible to rectify this issue with the existing dataset. Given the recency of CKM emerging as a topic of research interest, I acknowledge the value of having these results in the literature as a basis for future research despite this limitation.

*Response:*

Thank you for the reviewer’s suggestion.

We agree that over such a long follow-up period, the CKM stage of all participants is likely to change over time. We have included this as a study limitation in the Limitation section of our manuscript.

Point 8
Noting the authors have defined CKM levels for the purpose of this research in Table S1, I think it remains important to identify each difference between the AHA's definitions and the definitions in this paper and to clearly explain why in each case. This could be in the main paper or in the supplementary materials.

*Response:*

We agree with the reviewer’s suggestion. We have emphasized the differences between the AHA’s definitions and the definitions used in this study in both the *Supplementary Materials* and the *Main Paper*.

Table S1 footnote:

In this study, the definition of CKM is based on the AHA’s criteria, but there are several key differences to note:

- The cut-off points for BMI and waist circumference in this study follow the AHA-defined *Asian criteria*.
- The definition of fasting blood glucose for CKM Stage 1 (100–124 mg/dL) is consistent with the AHA definition. However, since HbA1c measurements are not available in this cohort, we did not include the criterion of HbA1c between 5.7% and 6.4%.
- The definition of CKD in CKM Stages 2–4 is based on the CKD Epidemiology Collaboration (CKD-EPI) formula, which aligns with the AHA definition. However, for proteinuria assessment, the original AHA definition uses albumin-to-creatinine ratio (ACR), whereas this study relies on dipstick testing.
- The definition of *Clinical CVD* in CKM Stage 4 in this study is based on self-reported medication use or seeking medical attention for coronary heart disease, heart failure, or stroke. This differs from the AHA definition, which requires a clinical diagnosis by physicians.

We have also included these clarifications in the main text of the manuscript.

**Page 8, Line 169**

Point 9
I don't the authors have quite understood my point. Blood pressure lowering medicines can be used for many indications outside of hypertension e.g., fluid retention, migraine prophylaxis, anxiety. This means it is risky to run the logic of "anyone who takes a blood pressure lowering drug has hypertension" because you will flag people using blood pressure lowering drugs for non-hypertension indications as having hypertension. Perhaps the best approach here is one of clarification and acknowledging this as a limitation? So, for example, rather than saying "Hypertension" in Table 1, say "On antihypertensive therapy" or make it clear in the table notes.

*Response:*

We agreed with this point and have acknowledging this as a limitation.

In Table 1, we have revised our heading about hypertension.

“Screened hypertension or on antihypertensive therapy was defined as a history of hypertension, the use of antihypertensive medication, or a systolic blood pressure of ≥130 mmHg or a diastolic blood pressure of ≥80 mmHg.”

In limitation,

**Page 16, Line 447**

“In addition, we acknowledged that anyone who takes a blood pressure lowering drug may not have hypertension. We defined hypertensive participants as screened hypertension or under antihypertensive therapy.”

Point 12
This is now OK and required no further action. That said, please take extreme care when relying on overlap or separation of confidence intervals for the purpose of "significance". Point 21 in this publication has a nice explanation <https://link.springer.com/article/10.1007/s10654-016-0149-3>

*Response:*

Thank you for this suggestion and reference.

Point 18
I think the authors missed the last sentence in my comment: "I think it's also worth being clear in the Introduction why questions of participant awareness are important to be considered as part of this particular study."

*Response:*

We have added the following explanation to the Introduction:

**Page 6, Line 111**

“We also assessed public awareness of each CKM component. In addition to evaluating awareness of diabetes and hypertension, we also examine CKD awareness in this study, particularly given that public awareness of CKD is generally lower compared to other CKM components.”

Point 19
Very minor but suggest using the word "associated" rather than "correlated" in this context because correlation is a specific statistical concept.

*Response:*

“We have revised accordingly.

**Page 14, Line 371**

“Although different risks contribute to various causes of death at varying levels, increased number of components was consistently associated with a higher risk of mortality (Table 3, Table S6-1 and S6-2).”

Point 20
Thank you for providing a definition for PAF in your methods. Please also ensure any results you want to discuss in the Discussion are presented first in the Results. This applies to all results, not just PAF.
*Response:*

We have ensure results like sensitivity analysis and PAF in the Discussion are presented in the results.

Reviewer #3: The authors have addressed my concerns properly.
Response:

Thank you for the comments.
