# Supplementary material for: Cardiovascular–kidney–metabolic syndrome and all-cause and cardiovascular mortality: A retrospective cohort study
Source: PLoS Med. 2025 Jun 26;22(6):e1004629. doi: 10.1371/journal.pmed.1004629 (PMC12200875; doi:10.1371/journal.pmed.1004629)
Supplement: Attachment — Submitted filename: Response_Editorial_20240421.docx [file pmed.1004629.s025.docx]

Response to Editor and Reviewers’ comments.

Comments from Reviewers:
Reviewer #1: The authors have addressed all my comments, and I am happy to recommend acceptance. Well done and congratulations.
Response:

We thank the Editor and reviewers for the valuable comments. All reviewers provided important and specific comments on the content of the manuscript.

Any attachments provided with reviews can be seen via the following link:


------------------------------------------------------------
Requests from Editors:
GENERAL
* Please confirm that your title complies with to PLOS Medicine's style. Your title must be nondeclarative and not a question. It should begin with main concept if possible. "Effect of" should be used only if causality can be inferred, i.e., for an RCT. Please place the study design ("A randomized controlled trial," "A retrospective study," "A modelling study," etc.) in the subtitle (ie, after a colon).

Response:

We have placed the study design in the subtitle.

Cardiovascular-Kidney-Metabolic Syndrome and Its Contribution to All-Cause and Cardiovascular Mortality: A Retrospective Cohort Study

* The funding statement should include: specific grant numbers, initials of authors who received each award, URLs to sponsors’ websites. Also, please state whether any sponsors or funders (other than the named authors) played any role in study design, data collection and analysis, the decision to publish, or preparation of the manuscript. If they had no role in the research, include this sentence: “The funders had no role in study design, data collection and analysis, decision to publish, or preparation of the manuscript.”

Response:

We have revised our funding statement to follow this rule.

**Page 19**

“Mr. Min-Kuang Tsai, the principal investigator, received research funding from the National Science and Technology Council (<https://www.nstc.gov.tw/?l=en>), under grant number NSTC 113-2314-B-038-024. Mr. Min-Kuang Tsai, the principal investigator, also received research funding from Taipei Medical University–Shuang Ho Hospital (<https://shh.tmu.edu.tw/indexEng.aspx>), under grant number 113FRP-20. This study is supported in part by the Taiwan Ministry of Health and Welfare Clinical Trial Center (MOHW110-TDU-B-212-124004). The funders had no role in study design, data collection and analysis, decision to publish, or preparation of the manuscript.”

* Data availability statement: If available, please also provide an email address that provides a clear point of contact for researchers interested in the data. Please update the metadata in the online submission form with the details provided on page 18.

Response:

**Page 19**

Researchers interested in obtaining the detail methodology codes and related data may contact Mr. Yuan Chieh Chuang at jackie_chuang@mjlife.com. The data used in this research were authorized by MJ Health Research Foundation (Authorization Code: MJHRFB2014001C). The MJ Health Research Foundation administered the MJ Health Survey Database and MJ BioData, and the data are available at <http://www.mjhrf.org/en/index.php?action=download&tid=3&cid=1>

* Please include the statement on code availability in the data availability statement.
Response:

We have included the code availability in the data availability statement. Please find the above response.

* Please include the completed STROBE checklist as Supporting Information. Please add the following statement, or similar, to the Methods: "This study is reported as per the Strengthening the Reporting of Observational Studies in Epidemiology (STROBE) guideline (S1 Checklist)."

Response:

We have added the statement in the Methods. “This study is reported as per the Strengthening the Reporting of Observational Studies in Epidemiology (STROBE) guideline (Supplement material 1).”

* Please ensure that all abbreviations are defined at first use throughout the text (including statistical abbreviations). Please also check figures and tables.

Response:

We have checked all the abbreviations again.

* Please review your text for claims of novelty or primacy (e.g. 'for the first time', ‘pioneering’) and remove this language.

Response:

We have revised such claims in the text.

* Please check that any use of statistical terms (such as trend or significant) are supported by the data, and if not please remove them.

Response:

We have checked these terms.

* Please ensure that tables and figures, including those in supplementary files, are appropriately referenced in the main text.

Response:

We have checked these tables and figures properly referenced in the text.

* Statistical reporting: Please revise throughout the manuscript, including tables and figures.
a) Please report statistical information as follows to improve clarity for the reader "22% (95% CI [13%,28%]; p</=)".
b) Please separate upper and lower bounds with commas instead of hyphens as the latter can be confused with reporting of negative values.
c) Please define statistical definitions at first use and repeat the abbreviated definitions (HR, CI etc.) for each set of parentheses.

Response: We have so revised.

* Since you are referring to "sex" throughout the manuscript, we suggest using the terms "male" and "female" instead of "men" and "women”. Please revise throughout.

Response:

We have changed the terms with “Male” and “Female”.

ABSTRACT

* Please confirm that your abstract complies with our requirements, including providing all the information relevant to this study type <https://journals.plos.org/plosmedicine/s/submission-guidelines#loc-abstract> 
Response: OK.

* Please ensure that all numbers presented in the abstract are present and identical to numbers presented in the main manuscript text.
Response: OK.

* In the Abstract, please include the important dependent variables that are adjusted for in the analyses.

Response:

We have included the adjusted variables in the abstract.

“Multivariate Cox proportional hazards models, adjusted for age, gender, educational level, smoking status, alcohol drinking status, and physical activity groups, were used to calculate hazard ratios (HRs).”

* In the last sentence of the Abstract Methods and Findings section, please describe the main limitation(s) of the study's methodology.

Response:

We have revised accordingly.

“The primary limitation is that our analysis relied on baseline measurements only, without accounting for longitudinal changes.”

* Please include the study design, setting, length of follow up, and baseline cohort demographics (e.g. average age, sex, race/ethnicity).
Response:

We have revised accordingly.

**Page 3, Line 9**

“We analyzed a retrospective cohort of 515,602 participants aged ≥20 years from a health screening program conducted between 1996 and 2017 in Taiwan. We assessed the associations of all-cause mortality, cardio-vascular disease (CVD) mortality, and cause-specific mortality with CKM stages and its components—hypertension, diabetes mellitus (DM), chronic kidney disease (CKD), metabolic syndrome, and hyperlipidemia. All participants were followed for a median of 16.5 years (interquartile range: 11.5, 21.2 years). Multivariate Cox proportional hazards models, adjusted for age, gender, educational level, smoking status, alcohol drinking status, and physical activity groups, were used to calculate hazard ratios (HRs). We used Chiang’s life table method to estimate years of life lost due to each CKM component. Among all participants, 257,535 (49.9%) were women. The majority of participants (n= 368,578 participants, (71.5%)) met criteria for CKM syndrome, with prevalence rates of 19.5%, 46.3%, 1.9%, and 3.8% for stages 1, 2, 3, and 4, respectively.”

* l.30: We don't think the word "longitudinal" is appropriate here, since you didn't collect data at multiple points over time.

Response:

We have deleted this word.

“In the large ~~longitudinal~~ cohort study, the prevalence of CKM syndrome and its components were associated with risks of all-cause mortality, CVD mortality, and ESKD.”

* l.31: We suggest removing the term "dose-response" as it does not seem appropriate in this context.
Response:

We have removed this term in the abstract.

AUTHOR SUMMARY

* l.52: It is impossible for the lay reader to understand what "two components" means because you have not provided an explanation of the syndrome and its components. Also, it is not possible to understand the magnitude of the number two if you do not explain that there are five components until after the results.

Response:

Thank you for this note. We have added the explanation as follows.

**Page 4, Line 55**

“We assessed mortality risk on the basis of the number of CKM components (hypertension, chronic kidney disease, diabetes, metabolic syndrome, and hyperlipidemia) present. CKM syndrome was prevalent in 71.5% of participants, with nearly 90% of those aged ≥55 years affected. Among all participants, one-fifth (22.7%) of the participants had at least two components, while one-ninth (11.7%) had at least three.”

INTRODUCTION

* Please ensure that the introduction ends with a clear description of the study question or hypothesis. We currently feel that the beginning and end of the last paragraph are somewhat repetitive.
Response:

We have removed the repetitive part at the end of the last paragraph.

METHODS AND RESULTS

* Please provide the ethical approval number and details on the consent procedure in the main text as done on page 18.

Response:

We have provided the details of ethical approval in the method section.

**Page 7, Line 137**
“This study was approved by the Institutional Review Board of Taipei Medical University (Approval Number: N202404037). All participants gave written consent to analysis their unidentifiable data before being included in this study.”

* l.140ff: Please consider moving Table S1 to the main text. We believe that the tabular presentation of the CKM stages is easier for the reader to follow.

Response:

We have moved this table to main text Table 1.

* l.149-152: Please revise for grammar and syntax.
Response:

We have revised the grammar for this sentence.

**Page 7, Line 157**

“Because our cohort is relatively young, the generalizability of the prevalence estimates may be affected. To account for this, we have included age- and education-adjusted prevalence estimates to better project the prevalence in this population.”

* l.225: When reporting ages, please ensure to provide a unit, such as years.
Response: We have revised accordingly.

In this study, life expectancy refers to the average expected remaining years of life for individuals at ages 30, 40, 50, 60, and 70 years.

* In general, we feel that the results section needs to be improved in terms of detail, clarity and consistency. Throughout, there is a lack of reference to relevant tables/figures and the main tables and figures are not discussed in sufficient detail. Often the focus is on the analyses presented in the supplementary files, while the main results are described in only one or two sentences. Please consider whether moving some of the figures presented in the Supplementary Information to the main text would aid the reader.

Response:

We agree with the editor’s suggestions and will revise the manuscript accordingly. We will also move the relevant supplementary information into the main text. Our goal is to ensure that readers can clearly understand our research findings, and we remain committed to making further adjustments as needed to improve the presentation of our results.

* l.248ff: “Participants with CKM syndrome were more likely to be male (58.7%), have an educational level of middle school or lower (27.5%), current smoker (25.2%), and consume alcohol regularly (10.6%).” – It seems that this statement is not entirely correct. According to Table 1 participants with CKM syndrome were more likely to be non-smokers and non-drinkers. Please revise.

Response:

We are trying to compare participants with CKM syndrome and those without CKM syndrome. We have revised this statement as follows.

**Page 11, Line 261**

“Participants with CKM syndrome, compared to those without the syndrome, were older (aged 60 or above: 15.0% vs. 1.7%), more likely to be male (58.7% vs. 28.3%), have a middle school education or lower (27.5% vs. 7.8%), be current smokers (25.2% vs. 17.1%), and consume alcohol regularly (10.6% vs. 4.7%). Additionally, physical inactivity was more common among those with CKM syndrome (48.9%) compared to those without CKM (55.9%).”

* Table 1/Figure 1: According to Table 1, we would have expected the prevalence of CKM to decrease with age (60+ years: 15.0%), whereas according to Figure 1, the prevalence increases with age. We believe that the calculations for Figure 1 are currently not 100% clear. Please check and revise and see our comment below.
* Figure 1: Please convert the stacked bar chart to another data representation, such as a table, and be sure to provide not only the percentages, but also the numerators and denominators. Please also add a unit for age (years).

Response:

We have added a table to current figure 1.

The percentages, the numerators and denominators were also provided. We hope this can make the calculations for Figure 1 clear.

* l.276: We suggest referencing Table S2 here before describing the results.

Response:
Agree. We have so revised.

**Page 11, Line 290**

To further assess survival differences (Table S1), we analyzed RMST. CKM Stage 0 serves as the reference, with an RMST of 23.395 years. CKM stage 1 showed a slight reduction in RMST (23.248 years; difference: -0.147, 95% CI: -0.19, -0.11), while CKM Stage 2 had a more pronounced decline (23.140 years; difference: -0.255, 95% CI: -0.29, -0.22).

* l.291ff: Please include a reference to the relevant table showing the results on life expectancy (we assume Table S7). We find the description of the life expectancy results to be oversimplified. The numbers you present on line 292/293 seem to present only the results for age 30 (is this correct?). Also, we don't think it's appropriate to present ranges that mix results for males and females when you haven't presented a combined analysis. Overall, we don't think these results are presented appropriately or in sufficient detail. Please revise.

Response:

Because life expectancy must be calculated from a specified starting age, we used age 30 as the reference point to estimate remaining life years. The life expectancy estimates at other starting ages, when combined with remaining life years, yielded similar results. Therefore, we focused on presenting the findings based on age 30.

We acknowledge that the current presentation may not be sufficiently clear, and we have accordingly revised this section for improved clarity.

**Page 12, Line 321**

“We evaluated the differential mortality risk associated with CKM by comparing the remaining life expectancy across different CKM stages and components separately for male and female participants (Table S5). Among males starting at age 30, those with CKM stages 1, 2, 3, and 4 had, respectively, 0.2 (95% CI: -0.03 to 0.39), 2.5 (2.36 to 2.55), 6.2 (5.03 to 7.38), and 7.0 (6.42 to 7.58) fewer years of life expectancy compared with those at CKM stage 0. For females at the same starting age, those with CKM stages 1, 2, 3, and 4 had 1.5 (1.36 to 1.62), 3.7 (3.55 to 3.93), 4.5 (4.24 to 4.77), and 8.0 (7.37 to 8.68) fewer years of life expectancy, respectively, compared to CKM stage 0. When considering the number of CKM components, male participants with 1, 2, 3, 4, and 5 components had 1.5 (1.36 to 1.62), 3.7 (3.55 to 3.93), 4.5 (4.24 to 4.77), 8.0 (7.37 to 8.68), and 13.8 (12.07 to 15.45) fewer years of life expectancy, respectively, compared with those with no CKM components. Among female participants, the corresponding reductions in life expectancy were 2.9 (2.73 to 3.01), 5.5 (5.19 to 5.75), 6.9 (6.38 to 7.45), 9.9 (8.89 to 10.87), and 16.3 (14.61 to 18.00) years. On average, each additional CKM component was associated with a reduction in life expectancy of 2.5 years in males and 3.0 years in females (Figure 3).”

* l.298ff: Please note that the results for mortality risk based on the number of CKM components are not very clear. For example: "Among participants aged ≥65 years with all five CKM components, 69.9% had at least two components, 43.0% had at least three components, and 21.5% had at least four components". Do you mean with data available on all five components or between baseline and endline assessment? If an individual has all five components, it does not make sense to say that the individual has at least two, three, or four components. Please revise the entire paragraph for clarity.
Response:

We are sorry for the confusion. We have revised this section.

**Page 13, Line 338**

“We assessed the distribution of the number of CKM components (hypertension, CKD, diabetes, metabolic syndrome, and hyperlipidemia) present in our cohort (Table S6). One-fifth (22.7%) of the participants had two or more CKM components. However, among those with any CKM component, two-fifths (44.4%) had two or more components. Furthermore, one-ninth (11.7%) of participants in the entire cohort had three or more CKM components. However, among those with any CKM component, the proportion with three or more components increased to one-fifths (22.9%). Among participants aged ≥65 years with any CKM components, the multimorbidity became more common, with 69.9% had two or more components, 43.0% had three or more components, and 21.5% had four or more components.”

* l.310ff: Since Table 3 is included in the main text, we think it's these results that should be discussed first in the Results section, rather than the results from Tables S6-1 and S6-2. Please revise.

Response:
We have revised this accordingly.

**Page 12, Line 305**

“For all-cause mortality, compared with the absence of all the five CKM components, the presence of each additional CKM component was associated with a 22% increase in the risk of death (HR: 1.22; 95% CI: 1.21, 1.23) and a 37% increase in the risk of CVD mortality (HR: 1.37; 95% CI: 1.35, 1.40). The increase in risk was even more pronounced for diabetes mortality, kidney-related mortality, and ESKD incidence. The aforementioned risk increased exponentially (Table 4 and Figure 2). The increase in risk was even more pronounced for diabetes mortality, kidney-related mortality, and ESKD incidence. The aforementioned risk increased exponentially (Figure 2).

We also examined the risk of each individual CKM components (Table S4-1 and S4-2). Participants with diabetes alone had the highest risk of all-cause and CVD mortality (HR: 1.86; 95% CI: 1.67, 2.06 for all-cause and HR: 2.42; 95% CI: 1.84, 3.18). Participants with hypertension alone had the risk of CVD mortality (HR: 2.40; 95% CI: 2.18, 2.65). Participants with CKD or diabetes had the highest risk of ESKD (HR: 16.35; 95% CI: 12.51, 21.36 among participants with CKD and HR: 43.77; 95% CI: 31.64, 60.56 among participants with diabetes).”

* l.312: It appears that the numerical results for CVD mortality in the main text do not match the results in Table S6-1 (HR 2.42, 95%CI [1.84, 3.18] versus HR: 2.40; 95% CI: 2.18, 2.65). It appears that you used the results for "hypertension only" in the main text. Please check and revise.

Response:

You are right. We have revised accordingly.

* l.316: Please ensure to call out the relevant Table(s).
Response:

Please find the response for * l.310ff.

* l.322: Again, since Figure 2 is one of the main figures in your manuscript, we feel that you should explain the results in more detail.

Response:

Figure 2 is the figure for the Table 4 values. We have revised the explanation as follows.

**Page 12, Line 305**

“For all-cause mortality, compared with the absence of all the five CKM components, the presence of each additional CKM component was associated with a 22% increase in the risk of death (HR: 1.22; 95% CI: 1.21, 1.23) and a 37% increase in the risk of CVD mortality (HR: 1.37; 95% CI: 1.35, 1.40). The increase in risk was even more pronounced for diabetes mortality, kidney-related mortality, and ESKD incidence. The aforementioned risk increased exponentially (Table 4 and Figure 2). The increase in risk was even more pronounced for diabetes mortality, kidney-related mortality, and ESKD incidence. The aforementioned risk increased exponentially (Figure 2).”

* l.324: Please see our earlier comments (l.291ff) and revise accordingly.
Response:

We have combined this part to earlier response. Please find the response for l.291ff.

* Table S8: Please explain the two groups for hypertension and ensure to describe the relevant group in the main text with sufficient detail.

Response:

Because in some regions the definition of hypertension is still based on a threshold of 140/90 mmHg, we adopted the CKM definition using 130/80 mmHg as the cutoff in this study. Therefore, we present both definitions here and have incorporated the corresponding data into the main text.

**Page 13, Line 349**

“We assessed participants’ awareness of their chronic conditions based on whether they had sought medical treatment or received medication. Awareness rates were 54.6% for diabetes, 22.1% for hypertension- 130/80 mmHg, 43.4% for hypertension- 140/90 mmHg and 3.3% for CKD. For hyperlipidemia, 2.9% were receiving medication for high triglycerides, and for metabolic syndrome, 38.3% were on medication for diabetes, hypertension, or high triglycerides (Table S7).”

* Table 1: Is there a reason you chose different age groups than in Figure 1? Why is there a tilde instead of a dash for BMI?
Response:

Figure 1 was designed to more comprehensively present the age-related changes across different CKM stages; therefore, we used 5-year intervals as age cutoffs. In contrast, Table 1 aims to highlight differences by age groups, and due to space limitations, different age cutoffs were used.

For the BMI section, we have replaced the tilde with a dash to ensure consistency.

* Table 2: Please add a footnote explaining that "All CKM" does not include stage 0. Why is kidney-related mortality the only group that includes "related"? Have you explained anywhere what 'heart disease' includes?
Response:

We have added a footnote, “All CKM" does not include stage 0” for all related tables.

We have added the definition for causes of death in the Method section.

**Page 8, Line 195**

“Causes of death were classified according to ICD-9 and ICD-10 codes and included all-cause mortality (ICD-9: 001–998; ICD-10: A00–Y98), CVD (390–459; I00–I99), heart diseases (410–414; I20-I25), stroke (430–438; I60-I69) and expanded cardiovascular diseases, which encompassed cardiovascular diseases plus diabetes (ICD-9: 250) and kidney diseases (ICD-9: 580–589). We included individuals diagnosed with ESKD as well as those with a kidney transplant.”

We have also revised “kidney-related mortality” to “kidney diseases mortality.”

* Table 3: Please see the comments for Table 2.
Response: We have revised accordingly.

* Figure 2: Please indicate in the figure caption the meaning of the points and whiskers. Please show the axis beginning at zero. If this is not possible, please show a break in the axis.

Response:
“The points and whiskers represent the hazard ratios and their 95% confidence intervals.”

Some of the axes use a logarithmic scale; therefore, they start at 1 instead of 0. A corresponding footnote has been added to the figure.

“The axes for diabetes mortality, kidney disease mortality, and end-stage kidney disease incidence are presented on a logarithmic scale; the axes start at 1 instead of 0.”

* Figure 3: Please add that Stage 0/0 components served as the reference and add a brief explanation about the different components.
Response:

We have revised accordingly.

“Stage 0 and 0 component served as the reference. CKM components included the presence of hypertension, chronic kidney disease, diabetes, metabolic syndrome, or hyperlipidemia.”

* Supplementary figures and tables: Please revise the supplementary figures and tables according to the above comments and make sure that the figure description is sufficiently detailed. In general, figures and tables should be self-explanatory on a stand-alone basis. Please revise accordingly.

Response:

We have revised the supplementary figures and tables accordingly.

DISCUSSION

* General guidance on organizing the Discussion: a short, clear summary of the article's findings; what the study adds to existing research and where and why the results may differ from previous research; strengths and limitations of the study; implications and next steps for research, clinical practice, and/or public policy; one-paragraph conclusion.

* l. 350, “In total, 72% of the participants had CKM.” – please note that this statement is repetitive of the prior paragraph.
Response:

We have removed this repetitive sentence.

* l.353: Please note that new results (e.g. Table S9) should not be presented in the Discussion. Please move them to the Results section, e.g. as a supplementary analysis.
Response:

We have put this section to Results.

**Page 13, Line 356**

“We compared the prevalence rates of CKM stages between the U.S. National Health and Nutrition Examination Survey (NHANES) [29] and our cohort across different age groups (Table S8). CKM stage 2 accounted for the majority of CKM cases, representing approximately 55% in NHANES and around 65% in our study cohort.”

* ll.362-364: Please see our earlier comment that this statement is not clear.

Response:

We have revised as follows.

“In this study, among participants with any of the five components, 44.4% had two or more components, 22.9% had three or more components, and 7.8% had four or more components.”

* l.373: Please note that you no longer need to cite relevant tables/figures in your discussion.

Response:

We have removed this citation.

* l.388ff: Again, please note that new results (e.g. Table S10) should not be presented in the Discussion. Please move them to the Results section, e.g. as a supplementary analysis.
Response:

We have moved these PAF to the Results section.

**Page 13, Line 361**

“After accounting for both CKM prevalence and associated mortality risks, we estimated the PAF of CKM syndrome to be 18.7% (95% CI: 15.8–21.7) for all-cause mortality and 55.0% (95% CI: 49.0–60.4) for cardiovascular mortality (Table S10).”

* l.392: Please avoid repeating numerical results in the Discussion.

Response:

We have removed these numerical results.

**Page 16, Line 431**
“In our analysis, CKD also independently contributed a significant risk to CVD mortality even when controlling for blood pressure (130/80 mmHg), blood glucose (<126 mg/dL), absence of metabolic syndrome, and triglycerides (<135 mg/dL).”

* l.413ff: We feel that there is too much repetition of presenting the results rather than discussing the results.

Response:

We have removed the repetition.

* l.435: Why do you only mention the risk of ESKD as a strength and not discuss these results earlier in the discussion? Given that Taiwan has high dialysis prevalence and incidence rates, we think you could discuss these results earlier.

Response:

We have moved this part earlier in the Discussion.

**Page 16, Line 444**

“In addition to causes of CVD mortality, CKD is also the leading risk factor for the development of ESKD. Incorporating CKD into the CKM definition not only captures its impact on cardiovascular outcomes but also highlights the importance of preventing progression to dialysis. This is particularly relevant for certain Asian countries, including Taiwan, where the prevalence and incidence of dialysis are among the highest in the world [43]. Emphasizing CKD prevention is of critical importance.”

* l.460ff: Please see the comments above and include these findings in the results section.
Response:

We have moved these section to results.

------------------------------------------------------------
General Editorial Requests

1) We ask every co-author listed on the manuscript to fill in a contributing author statement. If any of the co-authors have not filled in the statement, we will remind them to do so when the paper is revised. If all statements are not completed in a timely fashion this could hold up the re-review process. Should there be a problem getting one of your co-authors to fill in a statement we will be in contact. YOU MUST NOT ADD OR REMOVE AUTHORS UNLESS YOU HAVE ALERTED THE EDITOR HANDLING THE MANUSCRIPT TO THE CHANGE AND THEY SPECIFICALLY HAVE AGREED TO IT.

Response:

We will follow all these rules.

2) Please ensure that the paper adheres to the PLOS Data Availability Policy (see <http://journals.plos.org/plosmedicine/s/data-availability>), which requires that all data underlying the study's findings be provided in a repository or as Supporting Information. For data residing with a third party, authors are required to provide instructions with contact information for obtaining the data. PLOS journals do not allow statements supported by "data not shown" or "unpublished results." For such statements, authors must provide supporting data or cite public sources that include it.
Response:

We have revised our Data availability statement to follow all these rules.

3) Please review your reference list to ensure that it is complete and correct. If you have cited papers that have been retracted, please include the rationale for doing so in the manuscript text, or remove these references and replace them with relevant current references. Any changes to the reference list should be mentioned in the rebuttal letter that accompanies your revised manuscript.
Response:

We have again reviewed our references.
__________________________________________________
In compliance with data protection regulations, you may request that we remove your personal registration details at any time.  (Use the following URL: <https://www.editorialmanager.com/pmedicine/login.asp?a=r>). Please contact the publication office if you have any questions.

Journal Requirements:

1) Please amend your detailed Financial Disclosure statement. This is published with the article, therefore should be completed in full sentences and contain the exact wording you wish to be published.
i). State what role the funders took in the study. If the funders had no role in your study, please state: “The funders had no role in study design, data collection and analysis, decision to publish, or preparation of the manuscript.”
ii). If any authors received a salary from any of your funders, please state which authors and which funders.
Response:

We have revised this part accordingly.

“Mr. Min-Kuang Tsai, the principal investigator, received research funding from the National Science and Technology Council (<https://www.nstc.gov.tw/?l=en>), under grant number NSTC 113-2314-B-038-024. Mr. Min-Kuang Tsai, the principal investigator, also received research funding from Taipei Medical University–Shuang Ho Hospital (<https://shh.tmu.edu.tw/indexEng.aspx>), under grant number 113FRP-20. This study is supported in part by the Taiwan Ministry of Health and Welfare Clinical Trial Center (MOHW110-TDU-B-212-124004). The funders had no role in study design, data collection and analysis, decision to publish, or preparation of the manuscript.”


2) Thank you for your statement " The Taiwan MJ Cohort is available to the worldwide research community and ofers opportunities for collaboration. Those applying for data access should contact the MJ Health Research Foundation at <http://www.mjhrf.org/en/index.php> " Please note that the contact for accessing your data cannot be an author on the manuscript. Please therefore replace the current contact information with a third party contact, such as an institutional data access committee.
Response:

We have replaced the contact information with a third party as follows.

“Researchers interested in obtaining the detail methodology codes and related data may contact Mr. Yuan Chieh Chuang at jackie_chuang@mjlife.com. The data used in this research were authorized by MJ Health Research Foundation (Authorization Code: MJHRFB2014001C). The MJ Health Research Foundation administered the MJ Health Survey Database and MJ BioData, and the data are available at <http://www.mjhrf.org/en/index.php?action=download&tid=3&cid=1>”
