# Supplementary material for: Cardiovascular–kidney–metabolic syndrome and all-cause and cardiovascular mortality: A retrospective cohort study
Source: PLoS Med. 2025 Jun 26;22(6):e1004629. doi: 10.1371/journal.pmed.1004629 (PMC12200875; doi:10.1371/journal.pmed.1004629)
Supplement: Attachment — Submitted filename: Editor_Comments_20250502.docx [file pmed.1004629.s026.docx]

Requests from Editors:

1) Title: We suggest changing the title to: Cardiovascular-Kidney-Metabolic Syndrome and All-Cause and Cardiovascular Mortality: A Retrospective Cohort Study
Response:

We agreed and have revised the title accordingly.

2) Please ensure to update the funding statement in the online submission form (i.e. the metadata) with the statement provided in the rebuttal.
Response:

We have updated the online submission form for the funding statement.

3) Please ensure to update the data availability statement in the online submission form (i.e. the metadata) with the statement provided in the rebuttal.
Response:

We have updated the online submission form for the data availability.

4) Thank you for providing your STROBE checklist. Please replace the page numbers with paragraph numbers per section (e.g. "Methods, paragraph 1"), since the page numbers of the final published paper may be different from the page numbers in the current manuscript.
Response:

We have revised our STROBE checklist.

5) Please remove any claims of novelty, e.g. on line 740 (“First, the study is a novel investigation…”).

Response:

We have revised this sentence.

“First, the study examines the relationship between CKM and various causes of mortality based on the AHA’s updated definition.”

6) The terms gender and sex are not interchangeable (as discussed in <https://www.who.int/health-topics/gender#tab=tab_1> ); please use the appropriate term.
Response:

We apologize for our initial misunderstanding of the difference between gender and sex. We have now revised all relevant content to use “sex” (male and female).

7) Please note that there are still instances of references to "men" and "women" instead of "male" and "female" instead of ". Please review and revise carefully (including tables and figures).
Response: We have revised these terms accordingly.

8) “Additionally, physical inactivity was more common among those with CKM syndrome (48.9%) compared to those without CKM (55.9%).” – Do you mean physical inactivity was more common among those without CKM syndrome?

Response:

We have revised this sentence.

“Moreover, the prevalence of physical inactivity was lower among participants with CKM syndrome (48.9%) than among those without CKM (55.9%).”

9) “We assessed the distribution of the number of CKM components (hypertension, CKD, diabetes, metabolic syndrome, and hyperlipidemia) present in our cohort (Table S6).” – Without presenting the relevant denominators, it is impossible for the reader to understand how the results were calculated. Please revise the table accordingly. We think it would be more informative to simply present the results of how many people had at least 1, how many had 2, how many had 3, etc. of the components.

Response:

Thank you for this suggestion.

We have provided the relevant denominators and revised this table accordingly.

“We assessed the distribution of the number of CKM components (hypertension, CKD, diabetes, metabolic syndrome, and hyperlipidemia) present in our cohort (N=515,602) (Table S6). One-fifth (22.7%) of the participants had two or more CKM components. However, among those with any CKM component (N=264,038), two-fifths (44.4%) had two or more components. Furthermore, one-ninth (11.7%) of participants in the entire cohort had three or more CKM components. However, among those with any CKM component, the proportion with three or more components increased to one-fifths (22.9%). Among participants aged ≥65 years with any CKM components, the multimorbidity became more common, with 69.9% had two or more components, 43.0% had three or more components, and 21.5%had four or more components.”

10) ll.308-312: “The increase in risk was even more pronounced for diabetes mortality, kidney-related mortality, and ESKD incidence. The aforementioned risk increased exponentially (Table 4 and Figure 2). The increase in risk was even more pronounced for diabetes mortality, kidney-related mortality, and ESKD incidence. The aforementioned risk increased exponentially (Figure 2).” - Please note that these are repetitive statements. Please revise.

Response:

We have removed the repetitive statements.

11) l.356, “We compared the prevalence rates of CKM stages between the U.S. National Health and Nutrition Examination Survey (NHANES) [29] and our cohort…” – To guide the reader, we think it would be helpful to add a short sentence about why you compared these two cohorts in an additional analysis. This also applies to the following paragraphs.
Response:

We have revised as follows.

As part of a comparison across different populations, we compared the prevalence rates of CKM stages between the U.S. National Health and Nutrition Examination Survey (NHANES) [29] and our cohort across different age groups (Table S8). CKM stage 2 accounted for the majority of CKM cases, representing approximately 55% in NHANES and around 65% in our study cohort.

After accounting for both CKM prevalence and associated mortality risks, we estimated the PAF of CKM syndrome to be 18.7% (95% CI: 15.8–21.7) for all-cause mortality and 55.0% (95% CI: 49.0–60.4) for cardiovascular mortality (Table S9).

To validate subgroup-specific patterns, the stratified analysis by sex and age groups still revealed a significant increase in the risk of all-cause mortality among those with CKM or more than two of its components (Table S10).

To evaluate consistency and potential changes over time, we conducted an analysis among participants who had undergone two health examinations. The average follow-up duration between these two examinations was 2.5 years (mean (SD): 2.5 (2.4)).
